# Efficient improvement of the proliferation, differentiation, and anti-arthritic capacity of mesenchymal stem cells by simply culturing on the immobilized FGF2 derived peptide, 44-ERGVVSIKGV-53

**DOI:** 10.1016/j.jare.2023.09.041

**Published:** 2023-09-28

**Authors:** Soo Bin Lee, Ahmed Abdal Dayem, Sebastian Kmiecik, Kyung Min Lim, Dong Sik Seo, Hyeong-Taek Kim, Polash Kumar Biswas, Minjae Do, Deok-Ho Kim, Ssang-Goo Cho

**Affiliations:** a Department of Stem Cell and Regenerative Biotechnology, Molecular & Cellular Reprogramming Center and Institute of Advanced Regenerative Science, Konkuk University, 120 Neungdong-ro, Gwangjin-gu, Seoul 05029, Republic of Korea; b Biological and Chemical Research Centre, Faculty of Chemistry, University of Warsaw, 02-089 Warsaw, Poland; c R&D Team, StemExOne Co., Ltd., 307 KU Technology Innovation Bldg, 120, Neungdong-ro, Gwangjin-gu, Seoul 05029, Republic of Korea; d Stem Cell Research Center of AMOLIFESCIENCE Co., Ltd, 91, Gimpo-daero 1950 Beon-gil, Tongjin-eup, Gimpo-si, Gyeonggi-do 10014, Republic of Korea; e Department of Biomedical Engineering, Johns Hopkins University, Baltimore, MD 21205 USA

**Keywords:** FGF-2, Peptide immobilization, Proliferation, Differentiation, A.I., Osteoarthritis

## Abstract

**Introduction:**

The stem cell microenvironment has been evidenced to robustly affect its biological functions and clinical grade. Natural or synthetic growth factors, especially, are essential for modulating stem cell proliferation, metabolism, and differentiation via the interaction with specific extracellular receptors. Fibroblast growth factor-2 (FGF-2) possesses pleiotropic functions in various tissues and organs. It interacts with the FGF receptor (FGFR) and activates FGFR signaling pathways, which involve numerous biological functions, such as angiogenesis, wound healing, cell proliferation, and differentiation.

**Objectives:**

Here, we aim to explore the molecular functions, mode of action, and therapeutic activity of yet undetermined function, FGF-2-derived peptide, FP2 (44-ERGVVSIKGV-53) in promoting the proliferation, differentiation, and therapeutic application of human Wharton’s jelly mesenchymal stem cells (hWJ-MSCs) in comparison to other test peptides, canofin1 (FP1), hexafin2 (FP3), and canofin3 (FP4) with known functions.

**Methods:**

The immobilization of test peptides that are fused with mussel adhesive proteins (MAP) on the culture plate was carried out via EDC/NHS chemistry. Cell Proliferation assay, colony-forming unit, western blotting analysis, gene expression analysis, RNA-Seq. analysis, osteogenic, and chondrogenic differentiation capacity were applied to test the activity of the test peptides. We additionally utilized three-dimensional (3D) structural analysis and artificial intelligence (AI)-based AlphaFold2 and CABS-dock programs for receptor interaction prediction of the peptide receptor. We also verified the in vivo therapeutic capacity of FP2-cultured hWJ-MSCs using an osteoarthritis mice model.

**Results:**

Culture of hWJ-MSC onto an FP2-immobilized culture plate showed a significant increase in cell proliferation (n = 3; *p < 0.05, **p < 0.01) and the colony-forming unit (n = 3; *p < 0.05, **p < 0.01) compared with the test peptides. FP2 showed a significantly upregulated phosphorylation of FRS2α and FGFR1 and activated the AKT and ERK signaling pathways (n = 3; *p < 0.05, **p < 0.01, ***p < 0.001). Interestingly, we detected efficient FP2 receptor binding that was predicted using AI-based tools. Treatment with an AKT inhibitor significantly abrogated the FP2-mediated enhancement of cell differentiation (n = 3; *p < 0.05, **p < 0.01, ***p < 0.001). Intra-articular injection of FP2-cultured MSCs significantly mitigated arthritis symptoms in an osteoarthritis mouse model, as shown through the functional tests (n = 10; *p < 0.05, **p < 0.01, ***p < 0.001, ****p < 0.0001), modulation of the expression level of the pro-inflammatory and anti-inflammatory genes, and improved osteochondral regeneration as demonstrated by tissue sections.

**Conclusion:**

Our study identified the FGF-2-derived peptide FP2 as a promising candidate peptide to improve the therapeutic potential of hWJ-MSCs, especially in bone and cartilage regeneration.

## Highlights

• Growth factors play important roles in stem cell proliferation and differentiation.
• Immobilization of FP2 peptide, 44-ERGVVSIKGV-53 is performed via EDC/NHS chemistry.
• FP2 peptide-cultured hWJ-MSCs show enhanced in vitro proliferation and differentiation.
• FP2 phosphorylates FRS2α and FGFR1 signaling pathways.
• Transplantation of FP2 peptide-cultured cells ameliorates arthritis symptoms in an osteoarthritis mouse model.

## Graphical abstract

**Figure:**
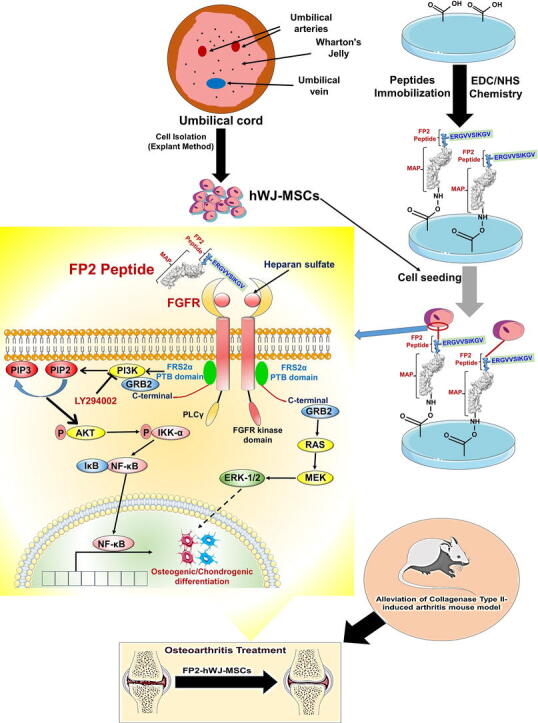

## Introduction

The field of regenerative medicine has grown rapidly in recent decades. The production of stem cells with high proliferation and differentiation capacities is critical for therapeutic applications and tissue regeneration [1]. Mesenchymal stem cells (MSCs) are multipotent stem cells with strong proliferation, self-renewal, and multilineage differentiation capacity [2]. The methods used to culture MSCs strongly influence their performance, characteristics, and clinical applications, and this is true even for MSCs purified from the same tissue source [3].

Various research groups have developed biomaterial-based culture systems for improving stem cell adhesion and proliferation for use in regenerative applications [4]. For instance, the E7 peptide (EPLQLKM), a peptide with a specific high affinity for bone marrow-derived MSCs (BM-MSCs), promoted the in vitro and in vivo homing of MSCs when covalently conjugated with polycaprolactone electrospun meshes [5]. Moreover, the immobilization of the E7 peptide in the collagen-binding domain of a collagen scaffold significantly improved MSC adhesion and infiltration and enhanced the wound-healing capacity of porcine skin [6].

The fibroblast growth factor (FGF) family is comprised of a broad spectrum of growth factors in vertebrates that play crucial roles in various key cellular functions, including survival, proliferation, differentiation, and migration [7], [8]. There are 18 polypeptides in the mammalian FGF family, and they range in size from 15 kDa to 38 kDa [9]. The canonical mammalian FGFs are grouped into six subfamilies based on sequence homology and phylogeny, including five paracrine subfamilies and one endocrine subfamily [10]. There are five paracrine subfamilies: the FGF-1 subfamily: FGF-1 and FGF-2; the FGF-4 subfamily: FGF-4, FGF-5, and FGF-6; the FGF-7 subfamily: FGF-3, FGF-7, FGF-10, and FGF-22; the FGF-8 subfamily: FGF-8, FGF-17, and FGF-18; and the FGF-9 subfamily: FGF-9, FGF-16, and FGF-20 [10]. The three members of the FGF19 subfamily (FGF19, FGF21, and FGF23) are considered endocrine signals.

FGF-2, which belongs to the FGF1 subfamily, is a key protein involved in the regulation of numerous biological functions related to the control of proliferation and differentiation in various cell lines [11], [12], [13], [14]. By binding to and activating the cognate cell surface tyrosine kinase receptors, FGF receptors (FGFRs), paracrine FGFs mediate biological actions [15], [16]. Transmembrane FGFR belongs to the tyrosine kinase receptor subfamily, and mammalian FGFR is encoded by four genes, namely FGFR1, FGFR2, FGFR3, and FGFR4 [16]. FGFR is composed of three extracellular immunoglobulin-like domains (Ig1, Ig2, and Ig3), a tyrosine kinase domain (in the cytoplasm), and a transmembrane domain (hydrophobic) [17].

Heparan sulfate (a sulfated glycosaminoglycan) is an essential co-receptor for the binding, dimerization, and ultimate activation of FGFR by paracrine FGFs [16], [18]. Upon activation of FGFR, the initiation of the intracellular signaling cascade is mediated by adaptor proteins, including the FGFR substrate (FRS)-2α and Sprouty proteins, which modulate the PI3K/AKT and RAS-MAPK signaling pathways [19], [20], [21]. Our research group previously reported the potent activities of various extracellular matrix (ECM)- and FGF-2-derived peptides on improving the proliferation, stemness properties, and adhesion of human pluripotent stem cells (hPSCs) [22].

The goal of our study was to devise a potent niche for the efficient proliferation and differentiation of human Wharton’s jelly (WJ)-derived MSCs (hWJ-MSCs), which is based on the immobilization of FGF-2 derived peptides onto the culture plate. To this end, we investigated the effects of various FGF-2-derived peptide mimetics (FPs), namely, canofin1 (FP1), a randomly selected undefined peptide (FP2), hexafin2 (FP3), and canofin3 (FP4). Previously, FP1, FP3, and FP4 peptides can mimic FGF-2 function during in vitro and in vivo applications when they interact with FGFR [23]. FP1 and FP4 have been shown to promote neuronal differentiation and exhibit neuroprotective activity for cerebellar granule neurons [24]. FP3 has been shown to promote the survival and growth of cerebellar granule neurons [25] and improve motor and cognitive functions in an R6/2 mouse model of Huntington's disease [26]. These peptides were conjugated with mussel adhesive proteins (MAP) to allow efficient orientation of the peptide mimetics on the culture plate, enable the peptide to correctly bind to the cells, and preclude non-specific binding [22], [27]. MAP, or Mytilus edulis foot proteins (Mefps) are secreted from the phenol glands of the mussel foot and are essential for byssus formation. MAP, an adhesive material secreted from the byssal threads and adhesive plaques of mussels, is environmentally friendly, biodegradable, biocompatible, efficiently attaches to coarse and wet platforms, and is rich in lysine and the catechol-containing 3,4-dihydroxy phenyl-L-alanine (DOPA) [28], [29]. The post-translationally modified amino acid DOPA, with its high isoelectric points, is present in all proteins with adhesive properties [30]. Various cell lines have been efficiently adhered to Mefps-coated culture plates, such as cancer cells [31], T-lymphocytes [32], neuronal cells [33], and osteoblasts and epiphyseal cartilage cells [34]. The immobilization of MAP-fused FGF-2 peptide mimetics onto the culture plate was carried out via chemical activation with ethyl dimethylaminopropyl carbodiimide (EDC) and N-hydroxy-succinimide (NHS) solutions [22], [35].

WJ-MSC, derived from gelatinous WJ of the umbilical cord tissue, is waste tissue after birth and possesses various merits, including outstanding proliferation and differentiation capacities, a high cell number, a reliable source of young MSCs, cost-effectiveness, and being easily isolated [36], [37], [38]. WJ-MSC possesses immunosuppressive properties due to their low expression of human leukocyte antigen (HLA) class I, which is low in WJ-MSC, and the lack of expression of HLA-DR [39], [40]. Of note, the use of the WJ-MSC application obviates the concern regarding the age of the donor and its consequences on cell quality and number [41], [42].

Our in vitro studies demonstrated hWJ-MSCs cultured on FP2-coated culture plates showed a significant increase in cell proliferation, self-renewal, stemness, pluripotency, and osteogenic and chondrogenic differentiation compared to the other tested peptide mimetics and control cells. Moreover, we detected high phosphorylation in the extracellular signal-regulated kinases (ERK) and protein kinase B (PKB) or AKT signaling pathways in FP2-cultured cells. Application of AKT signaling inhibitor significantly abrogated FP2-mediated enhancement of cell proliferation and differentiation. Furthermore, our in vivo studies demonstrated the possible therapeutic activity of peptide mimetic-cultured hWJ-MSCs in a collagenase type II (COL II)-induced experimental osteoarthritis (OA) mouse model, which shown in improving behavioral tests and the suppression of the expression levels of arthritis-related genes.

We also attempted to delve into the structural interactions of the studied peptides with the FGFR1 receptor via state-of-the-art structure prediction tools. We demonstrated differences in the receptor interaction among the peptides. To the best of our knowledge, this is the first study to demonstrate the positive impact of FGF-2-derived peptide mimetic FP2 on hWJ-MSC characteristics and therapeutic value.

## Materials and methods

### Cell culture and chemicals

In this study, hWJ-MSCs were used to evaluate tested peptides. Isolation of hWJ-MSCs that were used in our study was carried out as described in our previous studies [43], [44]. Cell culture was carried out using a culture medium composed of alpha-minimum essential medium (α-MEM) (Gibco, Waltham, MA, USA) supplemented with 10 % fetal bovine serum (FBS; Hyclone, Logan, UT, USA) and 1 % penicillin/streptomycin (P/S; Gibco), and then incubated in a humidified atmosphere at 37℃ and 5 % CO_2_.

To test the effects of ERK and AKT signaling pathways on the proliferation and differentiation of peptide-culture cells, we used LY294002 (40 µM, Sigma-Aldrich, St. Louis, MO, USA), which is an inhibitor of the PI3K/Akt pathway and PD98059 (30 µM, Sigma-Aldrich), which is an inhibitor of the MEK/ERK1/2 pathway, and the cultures were treated with these inhibitors for 48 h.

### Cell growth and doubling kinetics assays and immunophenotypic analysis

In vitro cell growth was estimated from P1 until cell growth was stopped via cell counting using the trypan blue exclusion assay. Cells were seeded onto a 35 mm plate at a density of 1x10^5^ cells and after each passage, they were harvested, stained with 0.4 % trypan blue solution, and counted using a hemocytometer under a phase-contrast microscope. Cell proliferation kinetics were measured based on cumulative population doubling (CPD) as reported previously [45], [46] and as follows: CPD = log (No. [harvesting]/no. [seeding]) log (2).

For immunophenotypic analysis, we used FACS analysis. The cells were detached using 0.25 % trypsin-EDTA (Gibco) and harvested via centrifugation (1500 rpm for 5 min). After removal of the supernatant, the cell pellet was suspended and incubated with primary antibodies diluted in Dulbecco-phosphate buffered saline (D-PBS)-containing 2 % FBS (1:200). The primary antibodies used in our study were CD90/Thy1 (Cat No. AF2067, R&D Systems, Minneapolis, MN, USA), CD105 (Cat No. MA5-11854, Invitrogen, Waltham, MA, USA), CD34 (Cat No. MAB72271, R&D Systems), and CD45 PD7/26/16 + 2B11 (Cat No. MA5-13197, Invitrogen). Cells were incubated with the primary antibodies for 1 h 30 min at 4℃, and then the cells were washed with D-PBS and centrifuged at 1200 rpm for 5 min. Next, the cells were incubated with the secondary antibodies, anti-mouse IgG Alexa Fluor 488 (Cat No. A11059, Invitrogen), and anti-Sheep lgG PE (Cat No. F0126, R&D Systems) for 1 h 30 min at 4℃ and then washed with D-PBS. Cell analysis was performed using a flow cytometer (BD Bioscience, San Jose, CA, USA) to estimate the fluorescence intensities produced by the labeled antibodies.

### Cell proliferation assay

This experiment aimed to estimate the effects of the AKT inhibitor, LY-294002, and the ERK inhibitor, PD98059, on the proliferation of FP2-cultured hWJ-MSCs. Cells were seeded in 96-well plates at a density of 4 × 10^3^ cells per well. After the incubation period, the cells were incubated with a medium containing 10 % CCK-8 solution for 2 h. Subsequently, the optical densities were measured at a wavelength of 450 nm.

### Multilineage differentiation

For multilineage differentiation, cells were seeded in a 24-well plate at a density of 2x10^4^ cells/well after reaching 80 % confluence. For osteogenic differentiation, we added an osteogenic induction medium containing Dulbecco’s modified Eagle’s medium–low glucose (DMEM-LG) (Invitrogen)) supplemented with 5 % FBS, 1 % P/S, 100 nM dexamethasone (Sigma-Aldrich), 50 μg/ml ascorbate-2-phosphate (Sigma-Aldrich), and 10 mM β-glycerophosphate (Sigma-Aldrich). We exchanged the differentiation medium with a fresh medium every three days for two weeks. We then validated the differentiation via the visualization of the accumulated mineralized calcium phosphate using alizarin red S staining after fixation with 4 % paraformaldehyde (PFA). We also detected the expression levels of osteogenic differentiation-associated marker genes, namely alkaline phosphatase (ALP) and bone gamma carboxyglutamate protein (BGLAP) using qPCR.

The chondrogenic induction medium contained DMEM-LG supplemented with 2 % FBS, 50 μg/mL ascorbate-2-phosphate, 100 μg/mL sodium pyruvate, 1 % insulin-transferrin-selenium-ethanolamine (ITS-X; Gibco), 100 nM dexamethasone, 40 μg/mL L-proline, and 10 ng/mL transforming growth factor-beta 1 (TGF-β1; Prospec, East Brunswick, NJ, USA). Alcian blue staining was used to validate differentiation by staining acidic mucopolysaccharides, such as glycosaminoglycans. The expression of chondrogenic differentiation-related marker genes including aggrecan and Sox9 was also assessed using qPCR.

### Peptide mimetics fabrication and immobilization

MAP-conjugated FGF-2 peptides were prepared as described previously [22]. The coupling reaction between EDC (AK Scientific, Union City, CA, USA) and NHS (AK Scientific) was required before coating the culture plate with MAP-binding peptides. A 10 mM EDC and 10 mM NHS mixture was prepared and dissolved in 20 mM sodium acetate buffer (pH 6.5). We added this mixture to the culture plate, ensured that it covered the entire surface of the plate, and then incubated it at room temperature (RT) for 30 min. After removal of the EDC/NHS mixture, we added MAP-FGF-2-derived peptides (0.05 μg/ml) and incubated them for 30 min at RT. Next, we removed the MAP-bound peptide motifs, washed them three times with distilled water, and dried them.

### Protein structure prediction

The 3D protein structures of the tested FGF-2-derived peptides were predicted based on their protein sequences obtained from the RCSBPDB database (http://www.rcsb.org) [47]. We used PyMOL software (www.pymol.org) to visualize the protein crystal structures, and the tested peptides were shown in assorted colors in the motif.

### Structure prediction of peptide-receptor interactions

Structure prediction of protein-peptide interactions was performed using state-of-the-art computational tools for protein-peptide docking and the AlphaFold neural-network-based method for structure prediction [48]. For the docking input, we used the FGF-2 protein structure (PDB ID: 1CVS, chain C) and peptide sequence. First, we employed two template-based docking techniques [49], [50], and, for the studied peptides, neither method found templates with significant similarity to the target complexes. Consequently, the predictions we obtained had low confidence scores. Next, we used the template-free docking method CABS-dock [51].

We used the AlphaFold2 neural network-based method [48] to predict the structures of peptide-FGF-2 complexes. In the Alphafold predictions, we used only sequence information (from the protein and peptides) and the ColabFold environment setup [52]. Finally, we prepared the contact maps for the interaction interfaces of peptides and receptors using Mapiya web server as reported [53].

### Colony-forming unit (CFU) assay

This assay was aimed at estimating the clonogenic, self-renewal, and proliferative capacities of MSCs. For this assay, a low density of cells (1x10^3^ cells) was plated onto 35-mm-diameter culture plates (Corning Life Sciences, Tewksbury, MA, USA) and cultured in a basic culture medium for two weeks. The cells were then washed twice with D-PBS and stained with 0.05 % crystal violet (Sigma-Aldrich) prepared in methanol for 30 min at RT. We then removed the crystal violet and rinsed the plate using D-PBS, followed by washing with distilled water. The plate was then dried and photographed, and colonies containing at least 50 cells were counted.

### RNA isolation, RT-PCR, and quantitative real-time PCR (qRT-PCR)

Total RNA was isolated using Labozol Reagent (LaboPass, CMRZ001, Cosmogenetech, Seoul, Korea), according to the manufacturer’s instructions, and the concentration was measured using a Nanodrop (ND1000) spectrophotometer (Nanodrop Technologies Inc., Wilmington DE, USA). Complementary DNA (cDNA) was synthesized from 2 μg of total RNA using an M−MuLV reverse transcription kit (Labopass, CMRT010, Cosmogenetech) and oligo Dt primers. PCR was performed using rTaq Plus 5x PCR Master Mix (EBT-1319 ELPIS Biotech, Daejeon, Korea), and the products were visualized using 1–2 % agarose gels. To quantify the changes in the expression levels of target genes, we carried out qRT-PCR using the Applied Biosystems 7500 real-time PCR system with SYBR green master mix (ELPIS Biotech).

RNA isolation from the mice bone tissue was carried out as described previously [54]. Before RNA isolation, we carefully cleaned the bone tissue from any adhered tissues or muscles or blood smears and then washed it three times with D-PBS. We then stored the one tissue at −80^ο^C until use. The tissue homogenization process was performed using mortar, pestle, and spatula that were chilled with liquid nitrogen. Using the chilled spatula, we transferred the frozen tissue to the chilled mortar and then we added liquid nitrogen over the tissue until the evaporation. The continuous tissue grinding with the chilled pestle with the addition of liquid nitrogen was carried out until obtaining a fine powder. We then added the Labozol Reagent to the pulverized bone tissue and then carried out RNA isolation and cDNA synthesis as described above. The expression levels of the target genes were normalized using the housekeeping gene GAPDH, and the relative expression was calculated using the comparative Ct method or ^ΔΔ^[55]. The sequences of the primers used in this study are listed in Table 1.

**Table 1:** List of primer sequences used for semi-quantitative RT-PCR.

**Gene**	**Primer**	**Species**	**Product size (bp)**	**ACCESSION No**
BGLAP	F-5′-CGC CTG GGT CTC TTC ACT AC-3′ R-5′-CTC ACA CTC CTC GCC CTA TT-3′	Homo sapiens	143	NM_199173.6
ALP	F-5′-CCA GGC TGG AGA TGG ACA AG-3′ R-5′-AGA TTT CCC AGC GTC CTT GG-3′	Homo sapiens	227	NM_001127501.4
Aggrecan	F-5′-CAC GAT GCC TTT CAC CAC GAC-3′ R-5′-TGC GGG TCA ACA GTG CCT ATC-3′	Homo sapiens	182	NM_001369268.1
Sox9	F-5′-TAA AGG CAA CTC GTA CCC AA-3′ R-5′-ATT CTC CAT CAT CCT CCA CG-3′	Homo sapiens	174	NM_000346.4
CCL7	F-5′-GAG ATC TGT GCT GAC CCC AC-3′ R-5′-CCA CTC TGA GAA AGG ACA GGG-3′	Homo sapiens	161	NM_006273.4
ENPP1	F-5′-GTC GTC AGT GGT CCT GTG TT-3′ R-5′-TGC AAA GGC GTC TGA GAT GT-3′	Homo sapiens	164	NM_006208.3
ITGA6	F-5′-CCT GCT GTT TTT GAC CAG CG-3′ R-5′-CAC TGT GAT TGG CTC TGG GA-3′	Homo sapiens	200	NM_001316306.2
C-Myb	F-5′-CAC AGA ACC ACA CAT GCA GC-3′ R-5′-CGA GGC GCT TTC TTC AGG TA-3′	Homo sapiens	161	NM_001130172.2
CCL20	F-5′-TTG CTC CTG GCT GCT TTG AT-3′ R-5′-GCC GTG TGA AGC CCA CAA TA-3′	Homo sapiens	133	NM_001130046.2
IL6	F-5′-GTC CAG TTG CCT TCT CCC TG-3′ R-5′-CTG AGA TGC CGT CGA GGA TG-3′	Homo sapiens	168	NM_000600.5
CDKN2B	F-5′-CAA CGG AGT CAA CCG TTT CG-3′ R-5′-ACA TCG GCG ATC TAG GTT CC-3′	Homo sapiens	129	NM_078487.2
GAPDH	F-5′-AAT CCC ATC ACC ATC TTC CAG-3′ R-5′-ATG ACC CTT TTG GCT CCC-3′	Homo sapiens	146	NM_001357943.2
ACAN	F-5′-TAC CCG GTA CCC TAC AGA GAC-3′ R-5′-ACA TTG CTC CTG GTC TGC AA-3′	Mus musculus	172	NM_007424.3
COL2A1	F-5′-CAT CTT GCC GCA TCT GTG TG-3′ R-5′-TGC CCC TTT GGC CCT AAT TT-3′	Mus musculus	163	NM_031163.3
SOX9	F-5′-CGT GCA GCA CAA GAA AGA CC-3′ R-5′-CTC ATG CCG GAG GAG GAA TG-3′	Mus musculus	171	NM_011448.4
IL-1RA	F-5′-AAG CAA CCA CCT TGA GCC TG-3′ R-5′-TGC AGG GTC TTT TCC CAG AAG-3′	Mus musculus	166	NM_001039701.3
IL-10	F-5′-GGT TGC CAA GCC TTA TCG GA-3′ R-5′-TTC AGC TTC TCA CCC AGG GA-3′	Mus musculus	116	NM_010548.2
TIMP2	F-5′-CAG GTA CCA GAT GGG CTG TG-3′ R-5′-CGC GCA AGA ACC ATC ACT TC-3′	Mus musculus	169	NM_011594.3
IL-6	F-5′-GTC CTT CCT ACC CCA ATT TCC A-3′ R-5′-TAA CGC ACT AGG TTT GCC GA-3′	Mus musculus	154	NM_031168.2
MMP13	F-5′-CTT CTG GCA CAC GCT TTT CC-3′ R-5′-ATG GGA AAC ATC AGG GCT CC-3′	Mus musculus	179	NM_008607.2
TMP1	F-5′-AGA TAC CAT GAT GGC CCC CT-3′ R-5′-TTG CAG AAG GCT GTC TGT GG-3′	Mus musculus	120	NM_001294280.2
TNFα	F-5′-GTA GCC CAC GTC GTA GCA AA-3′ R-5′-ACA AGG TAC AAC CCA TCG GC-3′	Mus musculus	137	NM_013693.3
GAPDH	F-5′-CTC ACT CAA GAT TGT CAG CA-3′ R-5′-GTC ATC ATA CTT GGC AGG TT-3′	Mus musculus	346	NM_001289726.1

### RNA-Seq and data analyses

We purified the total RNA from the tested samples using Labozol Reagent to prepare RNA libraries. Then, 1 µg of the total RNA was used to prepare the mRNA sequencing library using the Illumina TruSeq A Stranded mRNA Sample Preparation kit, as described in the manufacturer’s guidelines (http://www.lascience.co.kr/).

### Protein-protein interaction (PPI) networks

To understand the molecular mechanisms of FP2 in MSC proliferation and differentiation, we predicted the key PPI for hWJ-MSCs cultured on FP2 mimetics. For this purpose, we used the STRING database version 10.5 (https://string-db.org/) [56]. PPI networks were then drawn using Cytoscape (version 3.6.1; http://www.cytoscape.org/) [57].

### Western blot analysis

We extracted the cellular proteins using a mixture of 100 mM Tris-HCl (pH 7.5), 1 % Triton X-100 (Sigma-Aldrich), 10 mM NaCl, 10 % glycerol (Amresco, Solon, OH, USA), 50 mM sodium fluoride (Sigma-Aldrich), 1 mM phenylmethylsulfonyl fluoride (PMSF) (Sigma-Aldrich), 1 mM p-nitrophenyl phosphate (Sigma-Aldrich), and 1 mM sodium orthovanadate (Sigma-Aldrich) and the cell lysates were centrifuged at 13000 rpm for 15 min at 4 °C. The supernatant was carefully collected and placed in a new tube, and protein quantification was carried out using the Bradford protein assay reagent (Bio-rad, Hercules, CA, USA).

The proteins (30 μg) were separated using 8–12 % sodium dodecyl sulfate–polyacrylamide gel electrophoresis (SDS-PAGE) and then transferred onto nitrocellulose membranes (Bio-Rad). Membrane blocking was performed using 5 % skimmed milk in Tris-buffered saline for 1 h, followed by incubation with the appropriate primary antibodies against total ERK (Cat No. B7074, Assay Biotechnology, Fremont, CA, USA), phosphorylated ERK (Cat No. 9101 s, Cell Signaling Technology, Danvers, MA, USA), total AKT (Cat No. CSB-PA000855, CUSABIO, Houston, TX, USA), phosphorylated AKT (Cat No. CSB-PA008120, CUSABIO), IKKα (Cat No. 11930, Cell Signaling Technology), FGFR1 (Cat No. NBP2-33784, Novus Biologicals, Centennial, CO, USA), phosphorylated FGFR1 (Tyr653, Tyr654) (Cat No. 44-1140G, Invitrogen) polyclonal Antibody, FRS2 antibody (Cat No. 3836–100, BioVision, Waltham, MA, USA), phosphorylated FRS2-alpha (Cat No. 3864S, Cell Signaling) or ACTIN (Cat No. sc-8432, Santa Cruz Biotechnology, Inc., Santa Cruz, CA, USA) overnight at 4 °C. Afterward, the membranes were incubated with secondary antibodies (anti-mouse or anti-rabbit IgGs), which were conjugated with horse radish peroxidase (HRP) (Santa Cruz Biotechnology) for 1 h at RT. The protein signals were visualized using an enhanced chemiluminescence kit (Amersham Biosciences, Piscataway, NJ, USA) and a ChemiDoc TM Imaging System (Bio-rad). The antibodies used in this study are listed in Table 2.

**Table 2:** Antibody list.

Antibody	Assay	Dilution	Company source	Catalog Number
CD34	FACS	1:200	R&D system	MAB72271
CD45 PD7/26/16 + 2B11-		1:200	Invitrogen	MA5-13197
CD73/NT5E		1:200	Invitrogen	RG235718
CD90/Thy1		1:200	R&D system	AF2067
CD105		1:200	Invitrogen	MA5-11854
anti-Mouse IgG Alexa Fluor 488		1:200	Invitrogen	A11059
anti-Sheep lgG PE		1:200	R&D system	F0126
PCNA (D3H8P) XP	Immunofluorescent staining	1:400	CST	13,110
Ki-67 (D3B5)		1:400	CST	9129
anti-rabbit IgG Alexa Fluor 488		1:200	Invitrogen	A11008
anti-rabbit IgG DyLight ^TM^ 549		1:200	Vector laboratories	DI-1549–1.5
P-AKT	WB	1:500	CUSABIO	CSB-PA008120
T-AKT		1:500	CUSABIO	CSB-PA000855
P-ERK		1:1,000	CST	9101 s
T-ERK		1:1,000	Assaybio	B7074
T-FGFR1		1:1,000	Novus Biologicals	NBP2-33784
P- FGFR1 (Tyr653, Tyr654)		1:1,000	Invitrogen	44-1140G
T-FRS2		1:125	Biovision	3836–100
P-FRS2-alpha		1:1,000	CST	3864S
IKKα		1:500	CST	3G12
β-actin		1:1,000	Santa cruz	sc-8432
HRP linked anti-rabbit IgG		1:2,000	CST	7074
HRP linked anti-mouse IgG		1:2,000	CST	7076

FACS: Fluorescence-activated Cell Sorting.

WB: Western blot

### Senescence associated-β-galactosidase (SA-β-gal) assay

This experiment aimed to evaluate the senescent changes in the late passage (P22) hWJ-MSCs after culture on FP2-coated culture plates. The SA-β*-*gal protocol was carried out as previously described [58]. Briefly, cells were seeded onto a 35 mm dish that was pre-coated with the tested peptides and incubated at 37^ο^C and under 5 % CO_2_ until they reached 80 % confluence. Cells were then washed with D-PBS and fixed with a mixture of 0.2 % glutaraldehyde (v/v) and 2 % formaldehyde (v/v), which was diluted in D-PBS buffer for 5 min at RT, followed by removal of the fixative solution and washing twice with D-PBS. Next, a freshly prepared SA-β-gal staining solution was added to the cells and incubated overnight at 37^ο^C and without exposure to CO_2_.

SA-β*-*gal staining solution was composed of 40 mM citric acid/Na phosphate buffer, 5 mM K3[Fe (CN)6], 5 mM K4[Fe (CN)6] 3H2O, 2 mM magnesium chloride, 150 mM sodium chloride, and 1 mg/mL X-gal, which was dissolved in distilled water. After staining, the cells were washed twice with PBS and once with methanol, then dried and protected from light at RT until photographed under a phase-contrast microscope.

### Immunofluorescent staining

For the immunostaining, after the end of the incubation, cells were washed three times with PBS and then fixed with 4 % PFA (Cat No. P2031, Biosesang) for 15 min at RT. The cells were then permeabilized with 0.3 % Triton X-100 for 30 min at RT. Next, the cells were blocked with 3 % bovine serum albumin (BSA; Cat No. BSAS 0.1, Bovogen Biologicals Pty Ltd., Australia) to avoid non-specific binding. Next, the cells were incubated overnight with the primary antibodies at 4 °C. The cells were washed with PBS and then incubated with the secondary antibodies for 1 h 30 min at RT. The primary antibodies were rabbit anti-PCNA (Cat No. 13110, Cell Signaling Technology) and rabbit anti-Ki-67 (Cat No. 9129, Cell Signaling Technology), and the secondary antibody was DyLight™ 549 Goat Anti-Rabbit IgG (Cat No. DI-1549–1.5, Vector laboratories, Burlingame, CA, USA) and Alexa Fluor 488 goat anti-rabbit IgG (Cat No. A11008, Invitrogen) which were diluted in 3 % blocking buffer. In a 1:200 dilution in a blocking solution, all antibodies were used. The cells were finally counterstained with 4′,6-diamidino-2-phenylindole (DAPI) diluted in VECTASHIELD® Antifade Mounting Medium (Vector Laboratories). We obtained the fluorescent pictures for the cells after the antibodies were bound using an inverted fluorescent microscope (Carl Zeiss LSM 800). The list of antibodies that were used in this study is enumerated in Table 2.

### Animal experiments

After we demonstrated the efficient in vitro effects of FP2-cultured hWJ-MSCs in promoting in vitro cell proliferation and differentiation, we sought to verify the application of these cells in alleviating OA in an experimental mouse model. Six-week-old BALB/c *nu/nu* mice (female, 20 ± 2 g) were purchased from the ORIENT BIO Animal Center (Seongnam-si, Korea).

To properly acclimatize the mice, they were housed for one week in a well-ventilated room with adjusted temperature and humidity and under a 12 h light/12 h dark cycle before the experiment. Food and water were provided ad libitum. The mice were divided into three groups as follows: (1) a COL II-treated group, (2) an FP2-hWJ-MSC-treated group, (3) an hWJ-MSC-treated group, and (4) a saline-treated group sham (n = 10 mice in each group).

### In vivo osteoarthritis (OA) induction and cell injection

The in vivo induction of OA was carried out using COL II as reported [59], [60]. Before OA induction, all mice were anesthetized via intraperitoneal injection of 60 mg/kg alfaxalone (Alfaxan; Careside, Gyeonggi-do, Korea). The anesthetized mice were then subjected to an intra-articular injection of 6 μl of 13U of COL II from *Clostridium histolyticum* (Worthington Biochemical Company, Freehold, NJ, USA) that was dissolved in saline into the knee joint via a 26G Hamilton syringe, as described previously [61], [62]. After the COL II injection, OA induction was confirmed by observing joint swelling and impaired gait, and joint swelling was measured using a digital caliper. The cells or peptide-cultured cells (1x10^5^ cells/10 μl D-PBS) were injected into the joint three days after OA induction.

### Rotarod test

In this experiment, we used a rotating rod that generated forced motor activity to assess disturbances in the mice’s gait, balance, and coordination. We placed the test mice on a rotarod (JD-A-07TSM, Jeung Do Bio & Plant Co., Ltd., Korea) and subjected them to an acceleration speed. Before starting the experiment, the mice were allowed to train for 5 min at a fixed speed of 4 rpm. The actual experiment started 1 h after training at a speed that increased from 4 to 40 rpm (the speed accelerated by 4 rpm every 30 s). Between the test trials, the mice were allowed to rest for 30 min to avoid exhaustion. The efficacy of the injected cells was evaluated by calculating the time between the first ride on the rotarod and the first failure to maintain a position on the top of the rod, and by counting the number of falls (failing frequencies).

### Histological and RT-PCR analyses of OA induction and cell therapy

On day 28 after OA induction, the mice were sacrificed, and the knee joint was collected and fixed using 4 % PFA in PBS, followed by rinsing with PBS to remove traces of PFA. Decalcification was carried out using 10 % ethylenediaminetetraacetic acid (EDTA), and then the joint was subjected to infiltration with 30 % sucrose at 4 °C until it sank to the bottom of the container. Afterward, the tissue was transferred into a mold containing Frozen Section Compound (FSC 22 Frozen Section Media, Leica Biosystem, Richmond, IL, USA) and the tissue was carefully oriented. The mold was then kept over dry ice for several minutes for equilibration and then kept at −80 °C until cryosection. For cryosection, samples were mounted on a specimen chuck with Frozen Section Compound and then loaded onto the cryostat object holder with adjustment of the holder blade relative to the sample. Then, the samples were sliced into sections of 10 μm thickness, transferred onto slides using forceps, and dried at RT.

We stained the sections with Safranin O/fast green to determine the degree of cartilage degeneration, which is correlated with staining intensity [63], [64]. In addition, we stained the cells with toluidine blue to measure the damage caused by GAG-associated cartilage changes [65].

Based on the staining results, we calculated the OARSI scores to categorize the severity of the OA based on the degree of cartilage deterioration [66], [67]. For instance, grade 0 indicates a sound cartilage surface, and grade 5 implies severe cartilage degeneration. In addition, we isolated RNA from the joint tissues and synthesized cDNA as described above, and then estimated the changes in the expression level of the pro-inflammatory and anti-inflammatory genes using mouse-specific primers (Table 1).

### Statistical analyses

All statistical analyses were performed using the GraphPad Prism (GraphPad Software, San Diego, CA, USA). Most experiments were performed in at least three independent trials. Data are shown as the mean ± SEM. For the statistical significance calculation, an unpaired two-tailed Student's t-test was applied for comparing two groups or comparing to the control. For the multiple comparisons, one-way ANOVA followed by Tukey's post hoc test was performed. In all figures, the statistical significance is indicated by the asterisks (*): *p < 0.05, **p < 0.01, ***p < 0.001, ****p < 0.0001.

### Ethics statement

All experiments involving animals were conducted according to the ethical policies and procedures approved by the Institutional Animal Care and Use Committee (IACUC) at Konkuk University (approval no.: KU20127).

## Results

### Structures of FGF-2-derived peptides and FGFR1

One of the goals of our study was to assess the impact of FGF-2-derived peptides, namely FP1 (canofin1), FP2 (undefined), FP3 (hexafin2), and FP4 (canofin3), on the proliferation and differentiation of hWJ-MSCs. We prepared recombinant FGF-2-derived peptides by fusing them with the C-terminus of MAP and then used *Escherichia coli* to produce these recombinant proteins (rMAP-ECMfp) using a technique previously reported by our research group [22]. These peptide mimetics represent FGF receptor (FGFR) agonists that could interact with FGFR [23], [68].

**Fig. 1:**
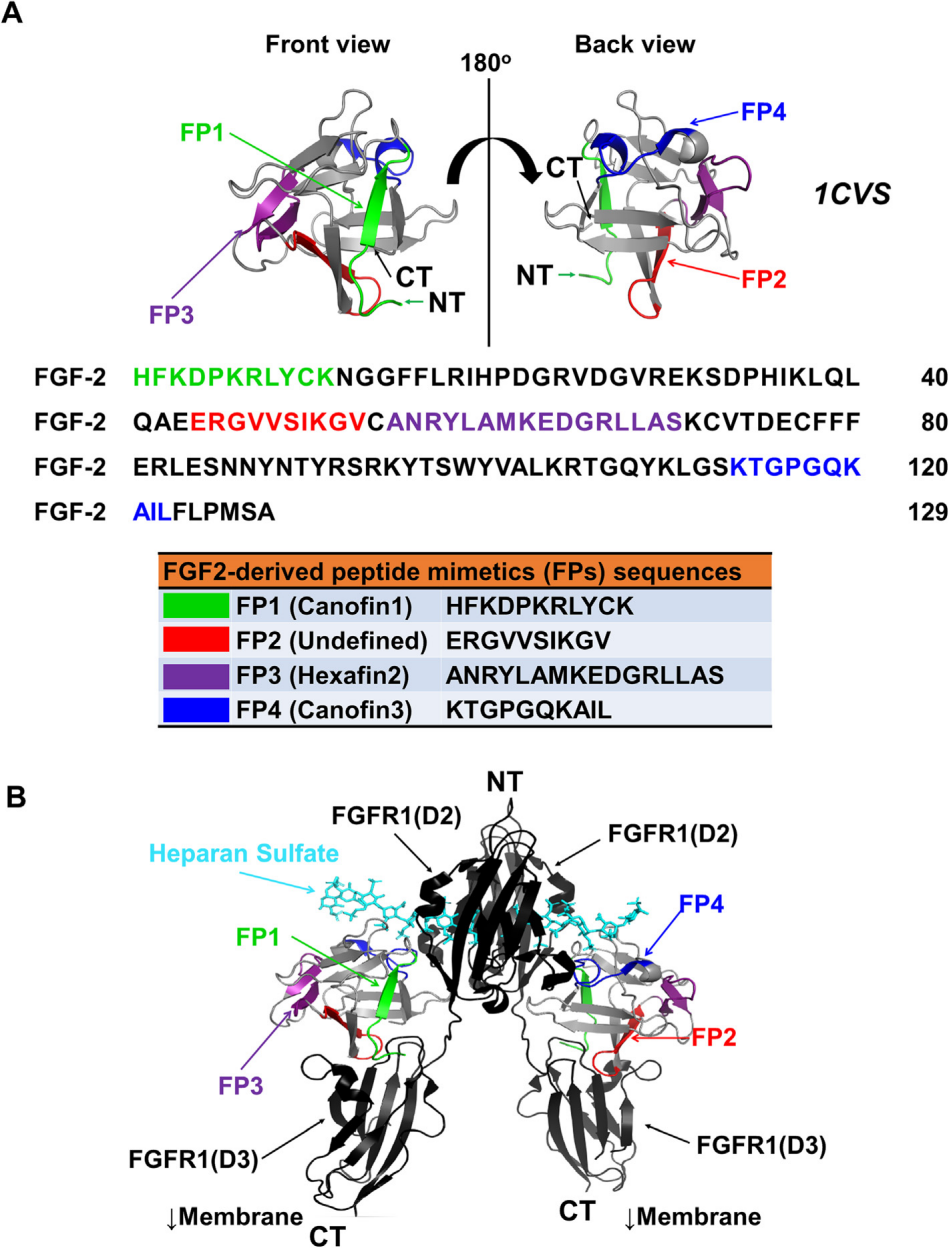
**3D structure of FGF2-derived test peptides and their specific modes binding to FGFR1. (A)** Front and back view of the 3D structure of FGF2 after rotation of 180^ο^ and with the test peptide mimetics marked in distinct colors. (**B**) The interactions between test peptides and FGFR1, with the test peptides indicated in assorted colors and heparan sulfate, which is an essential co-receptor for the efficient binding of FGF2 and FGFR. The figure shows the interaction between FGF2-derived peptides and FGFR (PDB ID: 1CVS). **Abbreviation**: CT, C-terminus; NT, N-terminus.

To predict the protein 3D structure of our tested peptides, we obtained their amino acid sequences from the RCSBPDB database (http://www.rcsb.org), as described by Rose et. al [47]. The amino acid sequences of the tested peptides are shown in Fig. 1**A**, in which each peptide is marked with a specific color.

We then visualized the 3D structures of the tested peptides using PyMOL Viewer (DeLano Scientific LLC, San Francisco, CA, USA). The FGF-2 structure, including the tested peptides (highlighted in color), was initially visualized, and then rotated at 180° (Fig. 1**A**). The interaction of the peptides with the extracellular immunoglobulin-like domains Ig2 (D2) and Ig3 (D3) of FGFR1 was also visualized (Fig. 1**B**).

### Structure prediction of FGF-2-peptide interactions

We also attempted to characterize protein-peptide interactions using state-of-the-art computational tools to study protein-peptide docking and the AlphaFold neural network-based method for structure prediction [48].

Docking tools can be divided into template-based docking techniques (using known experimental structures with a similar interaction interface, if they exist) and template-free docking methods that do not require any binding site information [69]. We used the FGF-2 protein structure (PDB ID: 1CVS, chain C) and peptide sequence as the input for the docking analysis. We employed two template-based docking techniques [49], [50] to study the peptides, but neither method found templates with significant similarity to the target complexes. Because of this, we obtained predictions that had low confidence scores. Next, we used a template-free docking method CABS-dock [51]. The CABS-dock results indicated that the peptides had a clear tendency to bind to specific regions within FGF-R1. This FGF-R1 region corresponded to the beta-sheet region near the extended loop of the C-terminal domain although different binding patterns were observed.

Finally, we used the AlphaFold neural-network-based method [48] for predicting the structures of the peptide-receptor complexes. In the first AlphaFold prediction run, we used the entire FGF-2 sequence (which consisted of two FGF-2 domains). We performed top-ranked AlphaFold predictions for the peptide-receptor complexes, as shown in **Supplemental** Fig. 1**A**, in which the peptides are colored, with the N-terminus in blue, the C-terminus in red, and the FGFR1 C-terminus in gray. **Supplemental Fig. 1B** illustrates the sequence of the FGF-2C-terminus that was used to generate the AlphaFold predictions. For the FP2 and FP3 complexes, the peptide fragments had high or moderate prediction confidence scores (**Supplemental** Fig. 1**C**). For both peptides, only a single binding mode was observed, which took place in the beta-sheet region near the extended loop of the C-terminal domain of FGF-2. This was the same binding region observed in the CABS-dock simulations. In contrast, for the FP1 and FP4 peptides, only low confidence scores were obtained for the peptide fragments. In the second AlphaFold prediction run, we limited our prediction to the C-terminal domain of FGF-2. Again, only a single binding mode was observed, and the top-scoring peptide models differed only in small local structural details. The obtained predicted local distance difference test (pLDDT) confidence scores were 70 for the central part of the FP2 peptide (regions with pLDDT around 70 are considered to be modeled well), while the terminal parts of FP2 had lower pLDDT values (which could indicate structural flexibility). Most of the FP3 peptide structures had residues with pLDDT > 90 (such regions are modeled with high accuracy). The top-ranked protein-peptide interaction interfaces for FP2 and FP3 with FGFR1 are presented in **Supplemental Fig. 1A**, which shows the contact interaction patterns for the presented models (**Supplemental** Fig. 1**C**). For the top-ranked protein-peptide models presented in **Supplemental** Fig. 1**A**, we also estimated the interaction energy parameters using the HADDOCK refinement in water [70].

Both the FP2 and FP3 models showed similar levels of favorable energy values for interactions (for electrostatic, desolvation, and van der Waals energy terms; see Table 3. However, the ligand efficiency measure (binding energy normalized by the molecular size) is likely to be significantly higher for FP2 than for FP3 (FP2 and FP3 have 10 and 16 residues, respectively), because one-third of the FP3 residues are engaged solely in internal beta-sheet interactions.

**Table 3:** Interaction energy parameters for theAlphaphold2 predictions of FP2-FGFR1 and FP3-FGFR1 complexes using the HADDOCK refinement in water. The table illustrates the most important numerical information for the top-ranked clusters. More details on FP2-FGFR1 and FP3-FGFR1 predictions is provided in the **Supplemental** Fig. 1**.**

	**FP2- FGFR1**	**FP3- FGFR1**
Van der Waals energy	−45.2 + 2.4	−52.3 + 1.2
Electrostatic energy	−65.7 + 8.5	−43.9 + 10.6
Desolvation energy	−13.7 + 0.9	−20.6 + 1.2
Buried Surface Area	1118.3 + 15.5	1226.4 + 11.4
HADDOCK score	−72.1 + 1.4	−81.7 + 1.0

HADDOCK scoring function consists of a simple linear combination of intermolecular van der Waals and Coulomb electrostatics energies and an empirically derived desolvation energy term [70]; despite its simplicity, this scoring function is consistent with the physico‐chemical properties of the modeled systems, encoding key aspects of biomolecular recognition. Finally, we visualized the intermolecular contact map for the interaction interfaces of FP2-FGFR1 and FP3-FGFR1 using the Mapiya web server [53], which illustrates the contact map between FGFR1 (protein-A) and FP2 and FP3 peptides (protein-B) as shown in **Supplemental** Fig. 1**D**. This data shows that both FP2 and FP3 interactions are stabilized by hydrophilic and hydrophobic contacts; however, the FP2-FGFR1 interaction is richer in hydrophobic contacts. Further analyses to correlate the hydrophobic interfaces with the peptide receptor interface are planned for further study.

### Screening the effects of FGF-2-derived peptides in enhancing proliferation and the differentiation of hWJ-MSCs

**Fig. 2:**
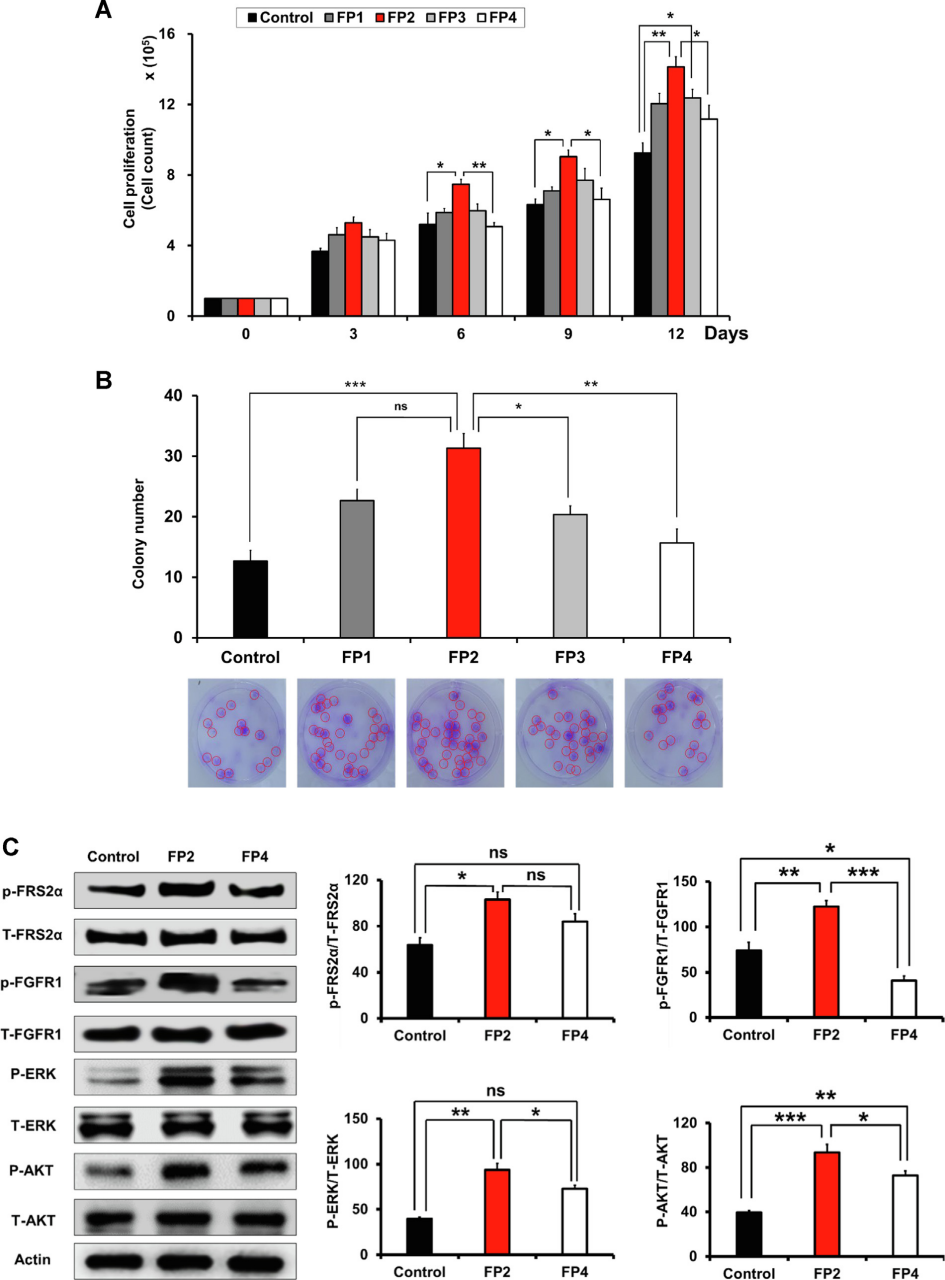
**Screening the impacts of FGF2-derived peptide mimetics on the proliferation, CFU capacities, differentiation, and the phosphorylation of AKT and ERK signaling in hWJ-MSCs.** (A) FP2-cultured MSCs showed the best proliferation capacity compared with other test peptides. Proliferation was estimated in a time-dependent manner up to day 12. (B) Similar to the proliferation results, culturing of MSCs onto FP2-coated plate showed the best CFU capacity, as confirmed by crystal violet staining. (C) The enhanced phosphorylation of ERK and AKT signaling pathways in FP2-cultured cells compared with FP4-cultured cells. The right panel represents graphic data from the western blot bands and shows the significant upregulation of ERK and AKT signaling with FP2. Data are expressed as mean ± SEM. For multiple comparisons of groups, a one-way analysis of variance (ANOVA) was performed followed by post hoc tukey’s multiple comparison. *p < 0.05, **p < 0.01, ***p < 0.001, ns, not significant.

To test candidate peptides, we used hWJ-MSCs, which were isolated as previously described by our research group [43], [44]. To determine how the peptides interacted with hWJ-MSCs, we first coated the culture plates with the test peptides via a coupling reaction between water-soluble EDC and NHS (**Supplemental** Fig. 7) as previously described [22], [35]. After peptide coating, hWJ-MSCs were seeded onto the coated plates, and alterations in cell proliferation were assessed in a time-dependent manner. Compared with the other tested peptides, only hWJ-MSCs cultured on FP2-coated plates showed a significant time-dependent increase in cell proliferation (Fig. 2**A**). In addition, the FP2 coating markedly improved the count of colony-forming units (CFU) of hWJ-MSCs as compared to the other tested peptides (Fig. 2**B** and **Supplemental** Fig. 2). Overall, the FP2 coating had the greatest effect in improving the proliferation and self-renewal capacities of hWJ-MSCs, whereas FP4 showed the least effect. Therefore, we selected FP2 and FP4 for further screening to assess their effects on the control of differentiation.

Similarly, FP2-cultured hWJ-MSCs showed a marked improvement in osteogenic differentiation, as shown by alizarin red staining (**Supplemental** Fig. 2**B**) and the higher expression of two osteogenic differentiation-related marker genes, namely ALP and BGLAP (**Supplemental** Fig. 2**C**), as compared to both the control cells and FP4-cultured cells. Moreover, FP2-cultured cells showed better chondrogenic differentiation activity, which was confirmed by stronger Alcian blue staining (**Supplemental** Fig. 2**D**) and higher expression levels of chondrogenic-associated marker genes, including aggrecan and Sox9 (**Supplemental** Fig. 2**E**).

### FP2 coating activates FRS2α and FGFR1 signaling pathways and the downstream signaling, the ERK and AKT in hWJ-MSCs

In the FGF signaling pathway, the adaptor protein FRS2α recruits multiple downstream signaling pathways, such as the ERK1/2 and PI3K/AKT pathways, to the FGFR kinase. Here, we aimed at investigation of the implication of the phosphorylation of FRS2α and FGFR1 and the consequent activation of ERK and AKT signaling pathways. Western blot analysis data demonstrate the significant phosphorylation of FRS2α and FGFR1 pathways in FP2-cultured hWJ-MSCs compared to FP4-grown cells (Fig. 2**C**).

For the downstream signaling pathways, we also demonstrated the marked increase in the phosphorylation levels of ERK and AKT signaling pathways in FP2-cultured cells (Fig. 2**C**), which have a similar pattern to FRS2α and FGFR1 results in Fig. 2**C**.

Collectively, our results verified proved the significant potency of FP2 peptide to phosphorylate the FRS2α and FGFR1 signaling and their downstream pathways, AKT and ERK signaling.

### FP2 promotes hWJ-MSCs proliferation and delays the senescence-related changes in hWJ-MSCs

**Fig. 3:**
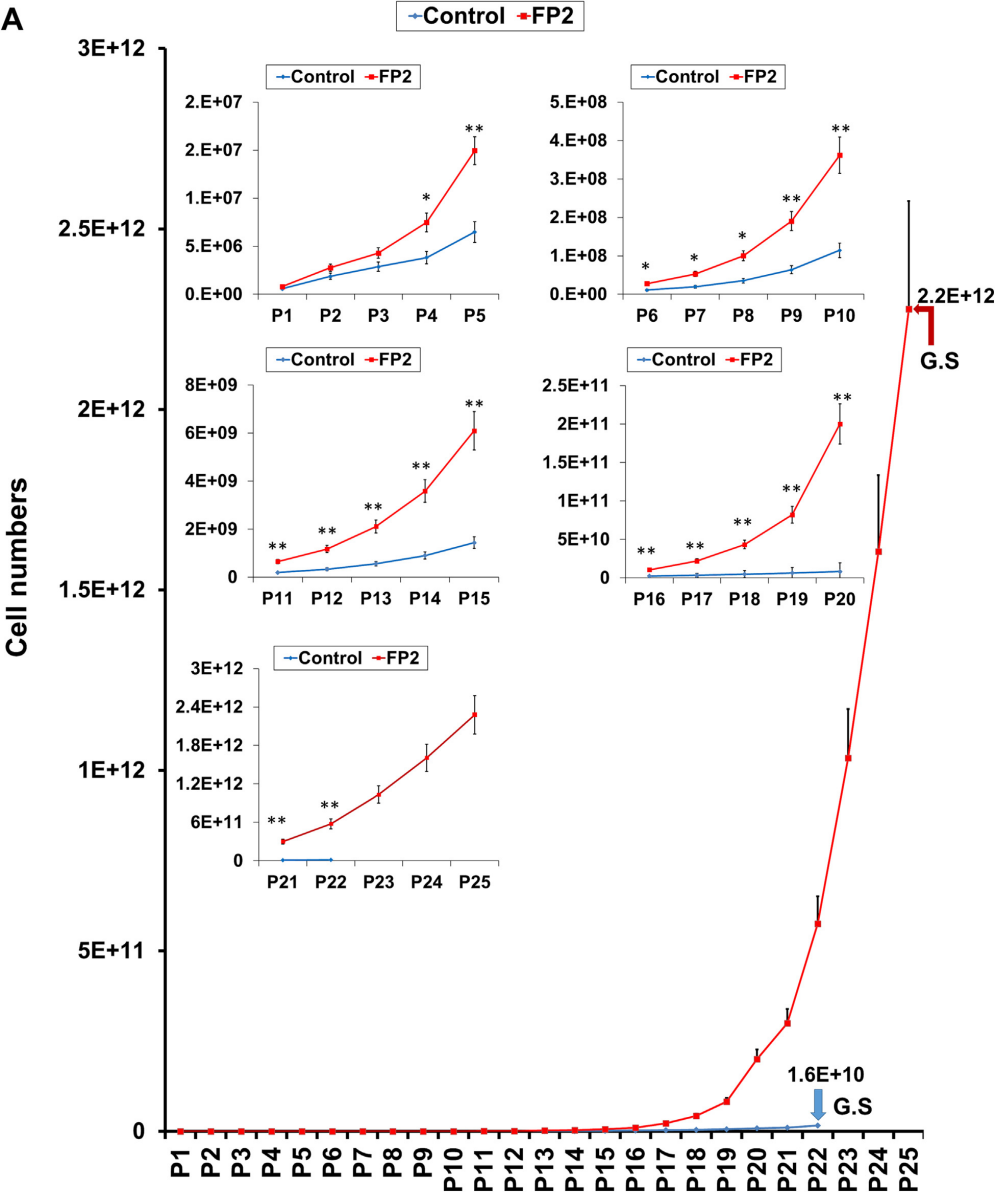

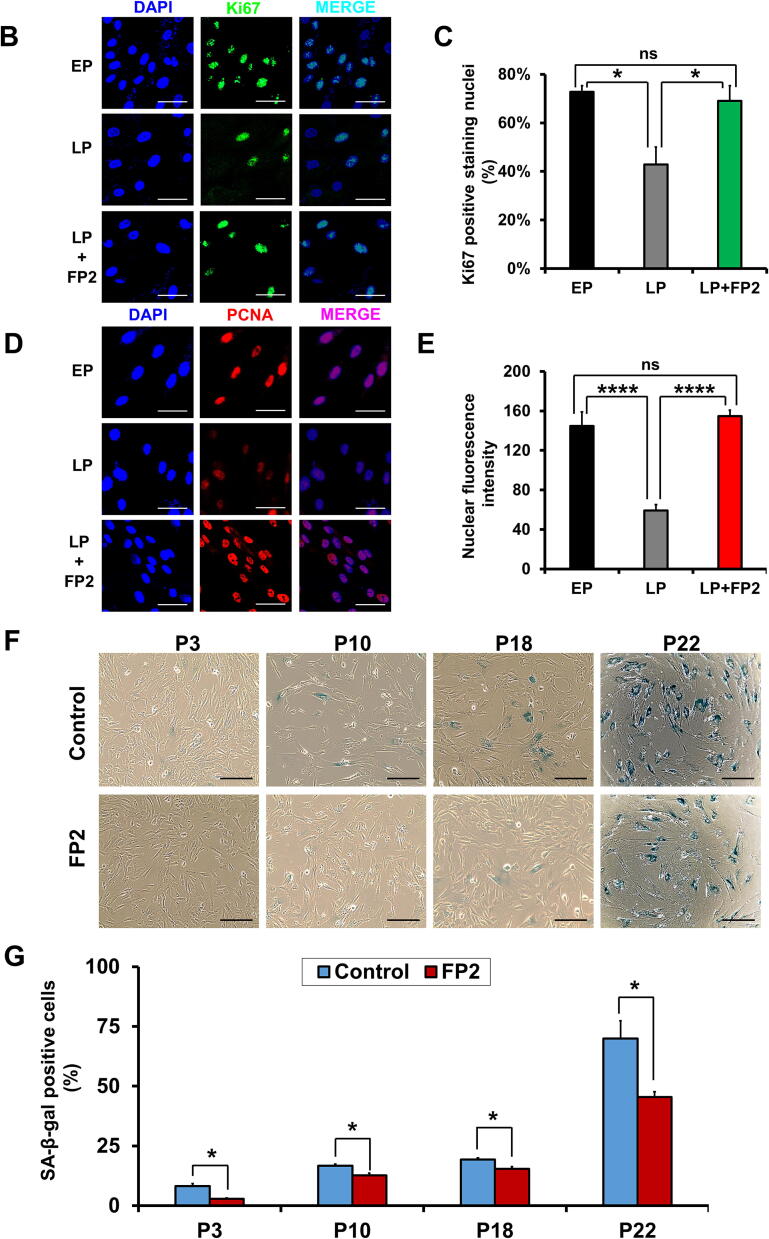
**The impacts of FP2 on the cumulative cell number, proliferation related marker and senescence-related changes of the hWJ-MSCs. (A)** FP2-cultured cells showed a significant increase compared with control uncoated cells. **(B)** Immunofluorescence analysis of Ki67 by confocal microscopy (nuclei are visualized using DAPI. The graphic data quantifying Ki67 expression is shown in **(C)**. **(D)** Immunofluorescence analysis of PCNA localization by confocal microscopy (nuclei are visualized using DAPI). The graphic data quantifying PCNA expression is shown in **(E)**. Scale bar, 50 μm. **(F)** Phase-contrast pictures show a passage-dependent increase in the senescence-related changes that are shown in the high number of SA-β-gal positive cells (blue colored), especially at P22 in hWJ-MSC, which improved in FP2-cultured cells. Scale bar, 250 μm **(G)** Graphic data representing the phase-contrast pictures for SA-β-gal staining. Data are presented as mean ± SEM. All experiments were performed three independent times. Statistical significance was determined using Two-tailed *t* test. For multiple comparisons of groups, a one-way analysis of variance (ANOVA) was performed followed by post hoc tukey’s multiple comparison. *p < 0.05, **p < 0.01, ***p < 0.001, ****p < 0.0001, ns, not significant. **Abbreviation**: G.S, Growth Stop; EP, Early Passage; LP, Late Passage; PCNA, proliferating cell nuclear antigen. (For interpretation of the references to color in this figure legend, the reader is referred to the web version of this article.)

We also analyzed changes in the growth kinetics of hWJ-MSCs grown on FP2-coated plates in a passage-dependent manner. Compared to control hWJ-MSCs cultured on uncoated plates, FP2-cultured cells showed a significant increase in cell proliferation starting at P4 and going up to P22 (Fig. 3**A**).

Interestingly, we detected a halt in the proliferation of the control cells after P22, whereas FP2-grown cells kept proliferating up to P25 (Fig. 3**A**). Similarly, we detected a significant decrease in the cell doubling time for FP2-grown cells compared to that of the control cells (**Supplemental** Fig. 3**A**). We also summarized the details of cell proliferation and population doubling time of FP2-cultured cells in comparison to the control cells in Table 4.

**Table 4:** Cell Cumulative Number and Doubling Time (Days).

**Passage**	**Cumulative Cell Numbers** **(**±SEM)		**Doubling Time** **(**±SEM**)**	
	Control	FP2	Control	FP2
P1	5.7.E + 05 ± 9.5.E + 04	8.1.E + 05 ± 1.1.E + 05	1.7 ± 0.13	1.6 ± 0.08
P2	1.9.E + 06 ± 3.1.E + 05	2.8.E + 06 ± 3.6.E + 05	2.4 ± 0.69	1.8 ± 0.52
P3	2.9.E + 06 ± 4.8.E + 05	4.3.E + 06 ± 5.6.E + 05	3.2 ± 0.71	2.5 ± 0.34
P4	3.8.E + 06 ± 6.4.E + 05	7.5.E + 06 ± 9.8.E + 05	3.5 ± 0.69	2.7 ± 0.37
P5	6.5.E + 06 ± 1.1.E + 06	1.5.E + 07 ± 2.0.E + 06	3.8 ± 0.04	2.8 ± 0.18
P6	1.1.E + 07 ± 1.8.E + 06	2.8.E + 07 ± 3.6.E + 06	3.8 ± 0.23	2.9 ± 0.06
P7	2.0.E + 07 ± 3.3.E + 06	5.3.E + 07 ± 6.9.E + 06	3.8 ± 0.13	2.9 ± 0.12
P8	3.5.E + 07 ± 5.9.E + 06	1.0.E + 08 ± 1.3.E + 07	3.9 ± 0.13	3.0 ± 0.08
P9	6.3.E + 07 ± 1.1.E + 07	1.9.E + 08 ± 2.5.E + 07	4.0 ± 0.08	3.0 ± 0.12
P10	1.1.E + 08 ± 1.9.E + 07	3.6.E + 08 ± 4.7.E + 07	4.0 ± 0.19	3.0 ± 0.15
P11	1.9.E + 08 ± 3.3.E + 07	6.5.E + 08 ± 8.5.E + 07	4.1 ± 0.04	3.0 ± 0.13
P12	3.3.E + 08 ± 5.5.E + 07	1.2.E + 09 ± 1.5.E + 07	4.2 ± 0.14	3.1 ± 0.13
P13	5.6.E + 08 ± 9.4.E + 07	2.1E + 09 ± 2.8.E + 08	4.4 ± 0.25	3.3 ± 0.19
P14	9.0.E + 08 ± 1.5.E + 08	3.6E + 09 ± 4.7.E + 08	4.5 ± 0.09	3.5 ± 0.04
P15	1.4.E + 09 ± 2.4.E + 08	6.1E + 09 ± 8.0.E + 08	4.5 ± 0.19	3.6 ± 0.04
P16	2.3.E + 09 ± 3.9.E + 09	1.0E + 10 ± 1.4.E + 09	4.8 ± 0.48	3.8 ± 0.57
P17	3.4.E + 09 ± 2.5.E + 09	2.2E + 10 ± 2.9.E + 09	5.0 ± 0.54	3.9 ± 0.15
P18	4.7.E + 09 ± 4.7.E + 08	4.3E + 10 ± 5.7.E + 09	5.4 ± 0.30	4.0 ± 0.09
P19	6.3.E + 09 ± 7.3.E + 08	8.2E + 10 ± 1.1.E + 10	5.6 ± 0.28	4.1 ± 0.46
P20	8.2.E + 09 ± 1.1.E + 09	2.0E + 11 ± 2.6.E + 10	5.8 ± 0.43	4.2 ± 1.40
P21	1.1.E + 10 ± 1.8.E + 09	3.0E + 11 ± 3.9.E + 10	6.0 ± 0.81	4.4 ± 0.97
P22	1.6.E + 10 ± 2.7.E + 09	5.8E + 11 ± 7.6.E + 10	6.3 ± 0.43	4.6 ± 0.18
P23	G.S	1.0E + 12 ± 1.3.E + 11	G.S	4.7 ± 0.58
P24	G.S	1.6E + 12 ± 2.1.E + 11	G.S	5.9 ± 0.62
P25	G.S	2.2E + 12 ± 3.0.E + 11	G.S	6.3 ± 0.72

We investigated whether there were any negative effects of FP2 peptide on the expression level of MSCs surface markers using FACS analysis and we did not detect any significant changes in surface marker expression levels in FP2-grown hWJ-MSCs (**Supplemental** Fig. 3**B**). We also demonstrated the positive impact of FP2 in enhancing MSCs proliferation in late passage (LP) cells via evaluation the expression level of the proliferation markers, Ki67 and Proliferating cell nuclear antigen (PCNA) using immunostaining. FP2-cultured LP WJ-MSCs showed a significantly high expression level of Ki67, which is comparable to the early passage (EP) WJ-MSCs (Fig. 3**B&C**) and PCNA proteins (Fig. 3**D&E**). Similarly, previous report demonstrated the role of FGF in boosting the expression of PCNA [71] and Ki67 [72]. PCNA is a nuclear protein that is highly expressed during the S/G2/M phases, weakly expressed during the G1 phase, and not expressed in cells during the G0 phase [73], [74]. The expression level of PCNA is extremely low in senescent cells [75].

Moreover, we sought to analyze the possible ability of FP2 coating to mitigate senescence-associated changes in hWJ-MSCs. We detected an increase in SA-β-gal staining of hWJ-MSCs at P22, and upon FP2 culture, we detected a marked decrease in the number of SA-β-gal-positive cells, which stained blue (Fig. 3 **F&G**).

### Analysis of differentially expressed genes (DEGs) in FP2-cultured hWJ-MSCs

**Fig. 4:**
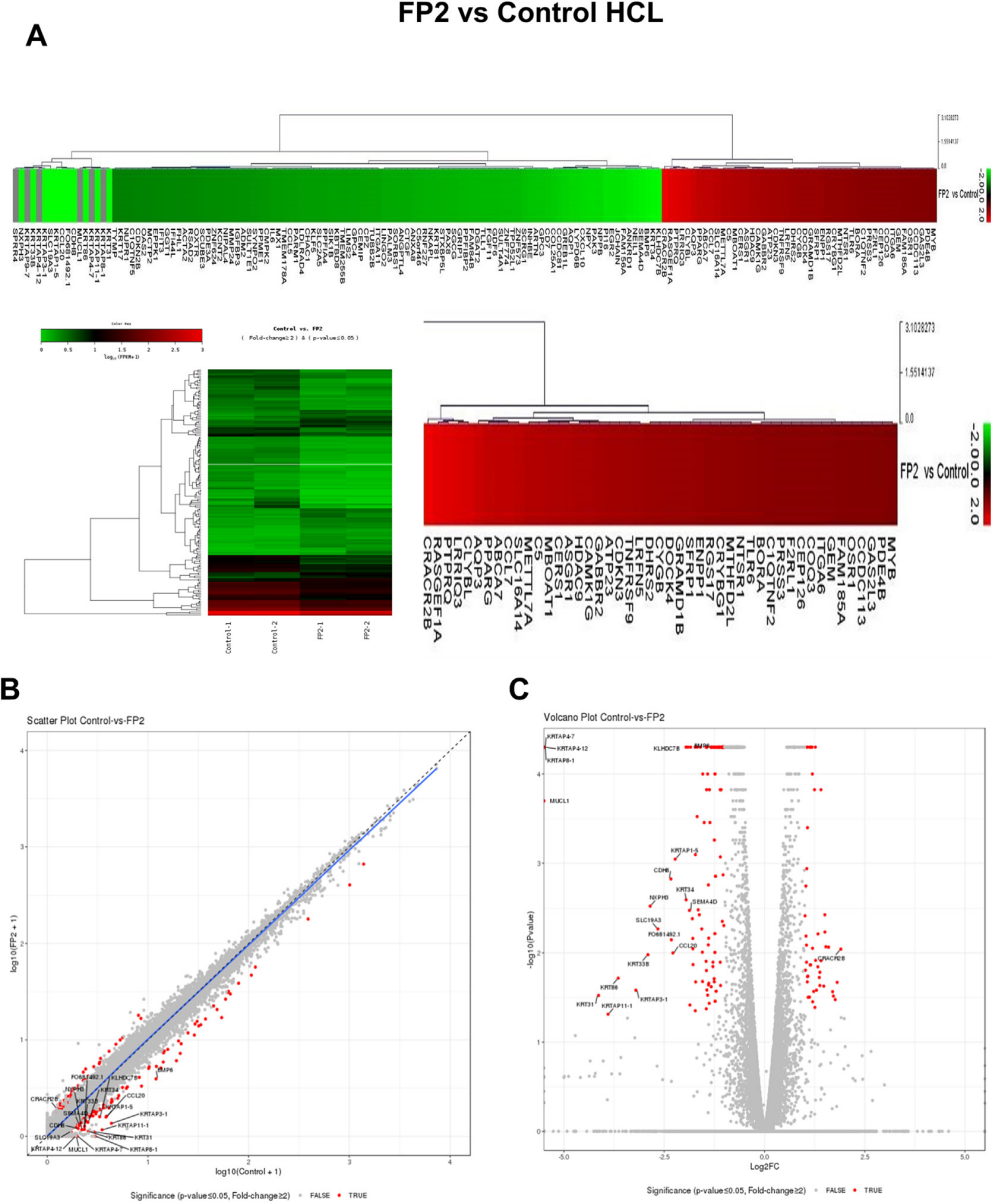

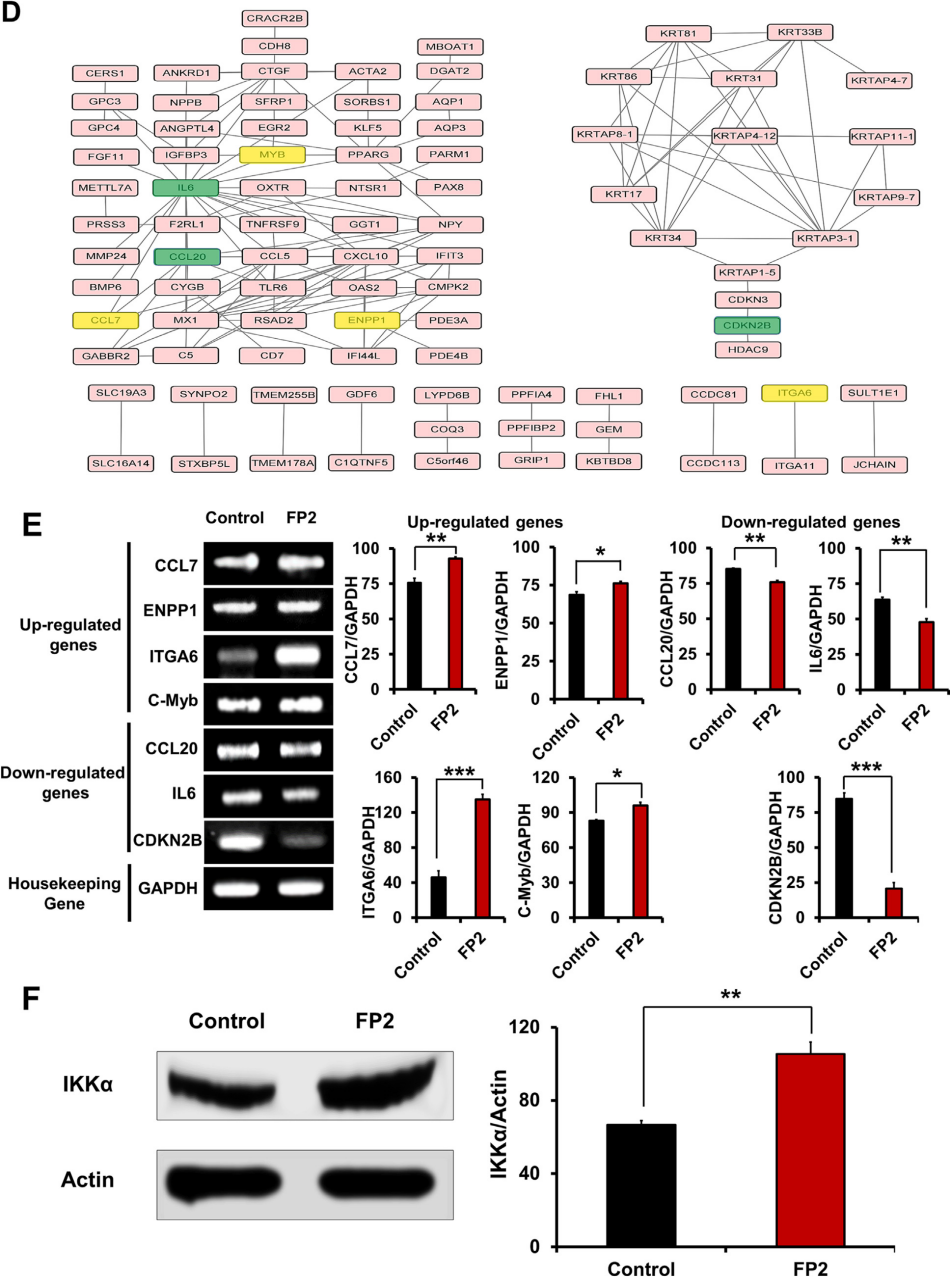
**Analysis of the differentially expressed genes (DEGs) in FP2-cultured cells versus control cells. (A)** Euclidean distance heatmap clustering representing significant DEGs produced using the MeV software. The green and red colors indicate decreased and increased gene expression, respectively. **(B)** Scatter plot for log_10_ (FPKM) values for comparing gene expression. **(C)** Volcano plot of DEGs, with log_2_ (fold change) versus -log_10_ (p-value). Red points indicate genes with a significant change in regulation, whereas black dots represent genes without a significant change. KEGG analysis identifying upregulated and downregulated signaling pathways in FP2-cultured cells versus control cells. **(D)** Protein-protein interaction networks showing overlapping DEGs between the FP2-cultured cells and control cells. The nodes and the lines indicate the DEGs and the interaction between them, respectively. Yellow boxes represent upregulated genes, and e green boxes represent down-regulated genes. **(E)** Validation of upregulated and downregulated DEGs using RT-PCR analysis (left panel). The right panel represents the graphic data obtained from PCR band analysis, as quantified using the ImageJ software. **(F)** Western blot analysis of IKKα, representing one of the upregulated DEGs. The right panel shows graphic data obtained from the western blot bands, which were quantified using ImageJ software. Statistical significance was determined using Two-tailed *t* test. *p < 0.05, **p < 0.01, ***p < 0.001. **Abbreviation**: CCL7, C–C motif chemokine ligand 7; ENPP1, ectonucleotide pyrophosphatase/phosphodiesterase 1; ITGA6, integrin subunit alpha 6; Myb, myeloblastosis; CCL20, C–C motif chemokine ligand 20; IL6, interleukin 6; CDKN2B, cyclin dependent kinase inhibitor 2B; GAPDH: glyceraldehyde-3-phosphate dehydrogenase. (For interpretation of the references to color in this figure legend, the reader is referred to the web version of this article.)

Our goal was to identify DEGs in FP2-grown hWJ-MSCs compared to control hWJ-MSCs using RNA sequencing (RNA-seq) analysis. We identified 158 DEGs with fold change ≤ 0.02 (47 upregulated genes and 111 downregulated genes) in FP2-cultured hWJ-MSCs versus control cells, as shown in the heatmap (Fig. 4**A**), scatter plot (Fig. 4**B**), and a volcano plot (Fig. 4**C**).

Next, we validated the functional interaction of the DEGs by using the Search Tool for the Retrieval of Interacting Genes (STRING) database to analyze the protein–protein interaction (PPI) networks. We identified 30 nodes and 62 edges among the DEGs analyzed using this database. Accordingly, we identified a group of genes with strong interactions, namely IL6, CCL7, and CCL20. IL6 interacted with Toll-like receptor 6 (TLR6), connective tissue growth factor (CTGF), and peroxisome proliferator-activated receptor gamma (PPARG) (Fig. 4**D**).

To characterize the biological functions of the identified DEGs, we performed data analysis using the Kyoto Encyclopedia of Genes and Genomes (KEGG), which showed the upregulation of the α6 integrin subunit (ITGA6), which is upstream of focal adhesion kinase (FAK) (**Supplemental** Fig. 4**A**). ITGA6, also known as CD49f, is one of the ITGA family and is a transmembrane glycoprotein adhesion receptor protein that is expressed more prominently in bone marrow MSCs (BM-MSCs) [76]. ITGA6 plays a key role in the modulation of self-renewal and stemness in stem cells [77], [78]. Additionally, we found the upregulation of the PI3K/AKT downstream signaling pathway, including C-Myb, which is related to cell survival (**Supplemental** Fig. 4**A**). c-Myb protein, which is a transcription factor, demonstrates the transition of dormant hematopoietic stem cells (HSCs) into homeostatic HSCs [79].

Using RT-PCR analysis, we confirmed the upregulation of several cytokine-cytokine receptor interaction-associated genes, namely chemokine (C–C motif) ligand 7 (CCL7) (related to chemokines), IL7B (interferon family), and 4-1BB (interferon family) (Fig. 4**E**). CCL7, also known as monocyte chemotactic protein-3 (MCP-3), is a chemokine that is abundantly produced by osteocytes and protects osteocytes against glucocorticoid-mediated cell death [80]. CCL7 and CXCL12 particularly modulate the migration of MSCs to assist in tissue regeneration after injury [81].

The high expression level of ITGA6, ectonucleotide pyrophosphatase/phosphodiesterase 1 (ENPP1), and C-Myb and the decreased expression of CCL20, IL6, and CDKN2B in FP2-cultured cells compared to the control cells was confirmed using RT-PCR analysis (Fig. 4**E**).

On the other hand, KEGG analysis showed the downregulation of TNF signaling pathway-related genes, such as CC15, CC120, CXC110, and IL6 (**Supplemental** Fig. 4**B**). Moreover, we again confirmed the downregulation of the expression levels of rheumatoid arthritis-associated genes, including IL-6, CCL20, and CCL5 using the RT-PCR analysis (Fig. 4**E**). To classify the DEGs based on biological process (BP), cellular components (CC), and molecular functions (MF), we performed gene ontology (GO) analysis using the database for annotation, visualization, and integrated discovery (DAVID) map. For BP, the most enriched GO terms were developmental process, cell differentiation, regulation of signaling, and tissue development (**Supplemental** Fig. 4**B**). Extracellular regions were mapped to the most enriched DEGs in CC (Fig. 4**I**). For MF, the most enriched GO terms were receptor ligand activity and receptor regulatory activity (**Supplemental** Fig. 4**C**).

In FP2-cultured cells KEGG analysis illustrates, the upregulation of PI3K/AKT downstream signaling, IκB kinase (IKK), which is upstream to nuclear factor-kappa B (NF-κB) and c-MYB that ultimately is associated with the cell survival (**Supplemental** Fig. 4**A**). As a part of showing the mechanism of FP2-mediated improved MSCs proliferation, we confirmed the increased phosphorylation of IKK-α using western blot analysis (Fig. 4**F**). IKK-α is one of the main upstream regulators of NF-κB [82]. In sum, FP2-cultured cells modulate key PI3K/AKT downstream elements including IKK-α and NF-κB that play essential roles in the enhanced cell proliferation.

### AKT inhibition abrogates FP2-mediated osteogenic and chondrogenic differentiation capacity

**Fig. 5:**
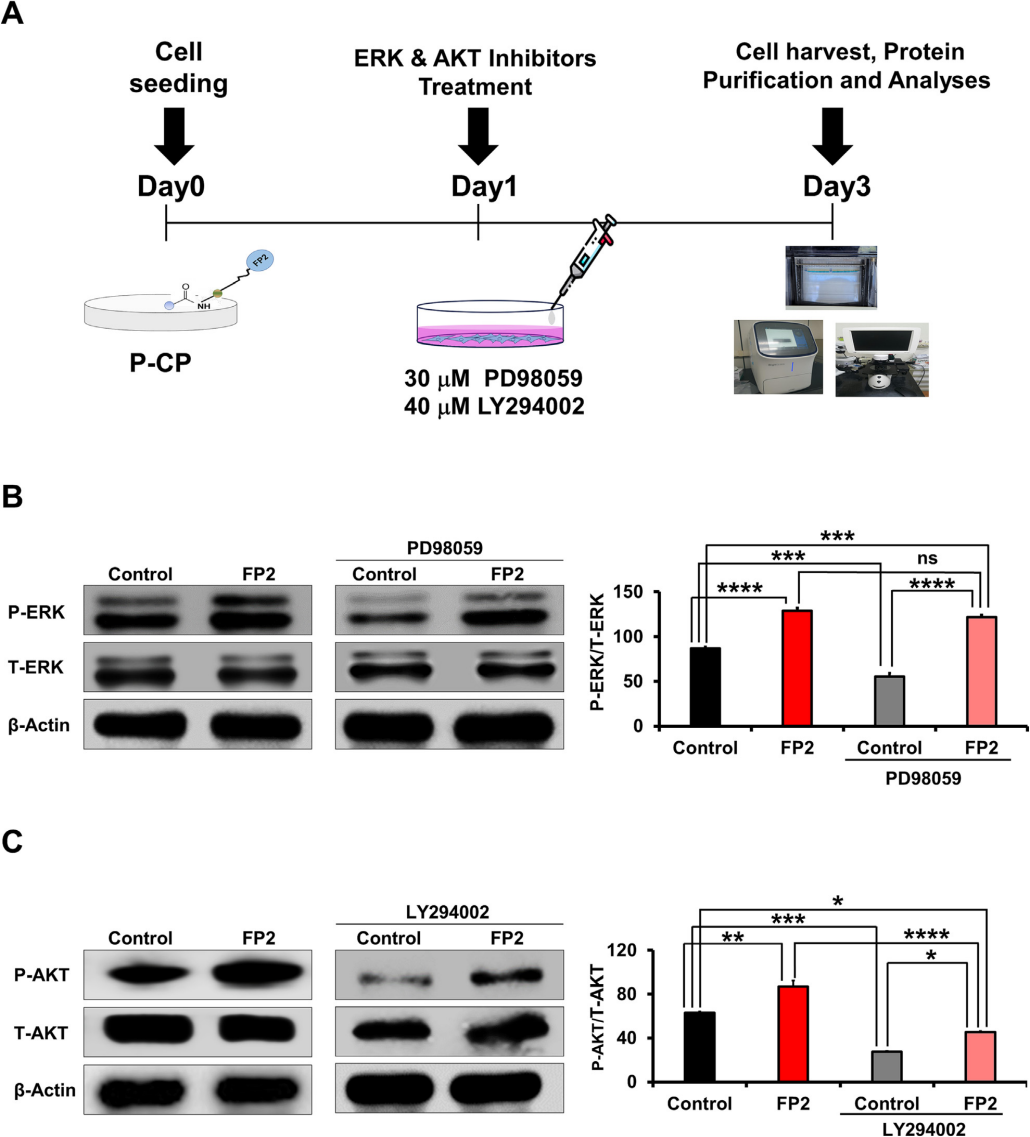
**Effects of AKT and ERK signaling pathways inhibitors on the FP2-cultured hWJ-MSCs. (A)** Schematic diagram illustrating the experimental plan for testing ERK and AKT signaling pathways inhibitors in FP2-cultured cells. Effect of an ERK signaling inhibitor, PD98059 **(B),** and an AKT inhibitor, LY294002 **(C)** on the phosphorylation in the ERK and AKT signaling pathways with and without FP2. The right panels indicate graphic data obtained from the western blot bands, which were quantified using the ImageJ software; **(B) &(C)**. Data are presented as mean ± SEM. For multiple comparisons of groups, a one-way analysis of variance (ANOVA) was performed followed by post hoc tukey’s multiple comparison, *p < 0.05, **p < 0.01, ***p < 0.001, ****p < 0.0001, ns, not significant. **Abbreviation**: P-CP, peptide coated plate.

RNA-seq results showed the impact of FP2 in modulating the PI3K/AKT signaling pathway and its downstream factors (Fig. 4 **& Supplemental** Fig. 4). Thus, we planned to investigate the protein expression level of AKT and ERK signaling pathways and apply their related inhibitors namely LY294002 and PD98059, respectively (Fig. 5**A**). Our results demonstrated the higher phosphorylation levels of the AKT and ERK signaling pathways in FP2-cultured cells compared with the control cells; however, AKT phosphorylation was higher than ERK phosphorylation (Fig. 5**B**). Application of AKT and ERK inhibitors marked suppression of phosphorylation levels, which recovered in FP2-cultured cells (Fig. 5**B**).

Next, we sought to determine the impact of AKT and ERK signaling pathway inhibitors on FP2-mediated enhanced osteogenic and chondrogenic differentiation capacities in hWJ-MSCs. We added these inhibitors during the differentiation time, and then we analyzed the changes in FP2 activity after the addition of each inhibitor (**Supplemental** Fig. 5**A**). Compared with the ERK inhibitor, the treatment of AKT inhibitor significantly abrogated FP2-mediated improved osteogenic differentiation in hWJ-MSCs, which was confirmed using alizarin red staining (**Supplemental** Fig. 5**B**). and quantitative PCR (qPCR) of the osteogenic differentiation-associated marker genes, ALP and BGLAP (**Supplemental** Fig. 5**C**). Similarly, the AKT inhibitor abolished the increased chondrogenic differentiation in FP2-cultured hWJ-MSCs, as verified by Alcian Blue staining (**Supplemental** Fig. 5**D**) and the analysis of the expression levels of the chondrogenic differentiation-related marker genes, aggrecan and Sox9 (**Supplemental** Fig. 5**E**). These results, together with the RNA-Seq analysis data, potently suggest that AKT signaling pathways are implicated in the FP2-mediated boosted proliferation and osteogenic and chondrogenic differentiation of hWJ-MSCs.

### Alleviation of OA symptoms in mice injected with FP2-cultured hWJ-MSCs

We aimed to test the effect of FP2-cultured hWJ-MSCs on the improvement of the inflammatory symptoms in experimental OA mouse model. Here, we performed an experimental OA mice model via the intra-articular injection of COLII, which cleaves type II collagen. This collagen type is the main macromolecular element of the extracellular matrix and is considered the main fibrillar collagen of the articular cartilage [83]. Moreover, type II collagen generates a fibrillar network and offers tensile strength by fending off swelling pressure brought on by the hydration of the matrix containing highly negatively charged proteoglycan aggregates. Therefore, the application of COLII for the proteolytic cleavage of type II collagen is one of the efficient strategies for creating a cartilage degeneration-associated arthritis model [84], [85], [86]. The matrix metalloproteinase (MMP) enzymes, in particular the collagenases interstitial collagenase (MMP-1), neutrophil collagenase (MMP-8), and collagenase-3 (MMP-13), which can all be produced by chondrocytes, have been proposed to be the main enzymes accountable for cleaving type II collagen during arthritis [87], [88].

Using a 26G Hamilton syringe (Hamilton Company, Reno, NV, USA), we injected 13U of COL II via intra-articular injection into the knee joint capsule. After five days, we routinely investigated OA-associated symptoms (**Supplemental** Fig. 6**A**) and injected the stem cells. The induction of OA was confirmed by the detection of a significant increase in the swelling of the joint capsule and rotarod device to determine whether the mice's balance, coordination, and gait are affected (**Supplemental** Fig. 6**A**).

**Fig. 6:**
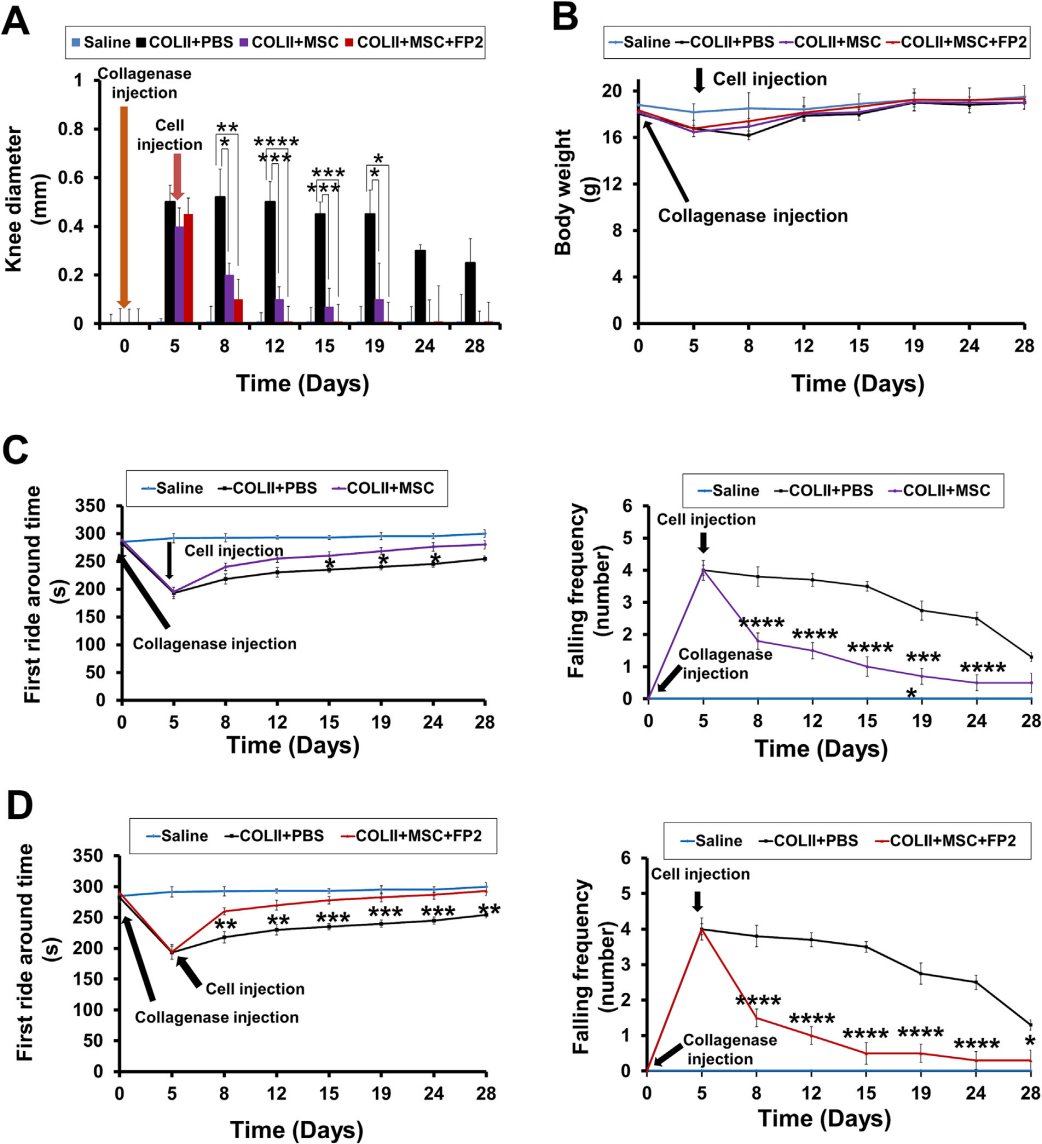

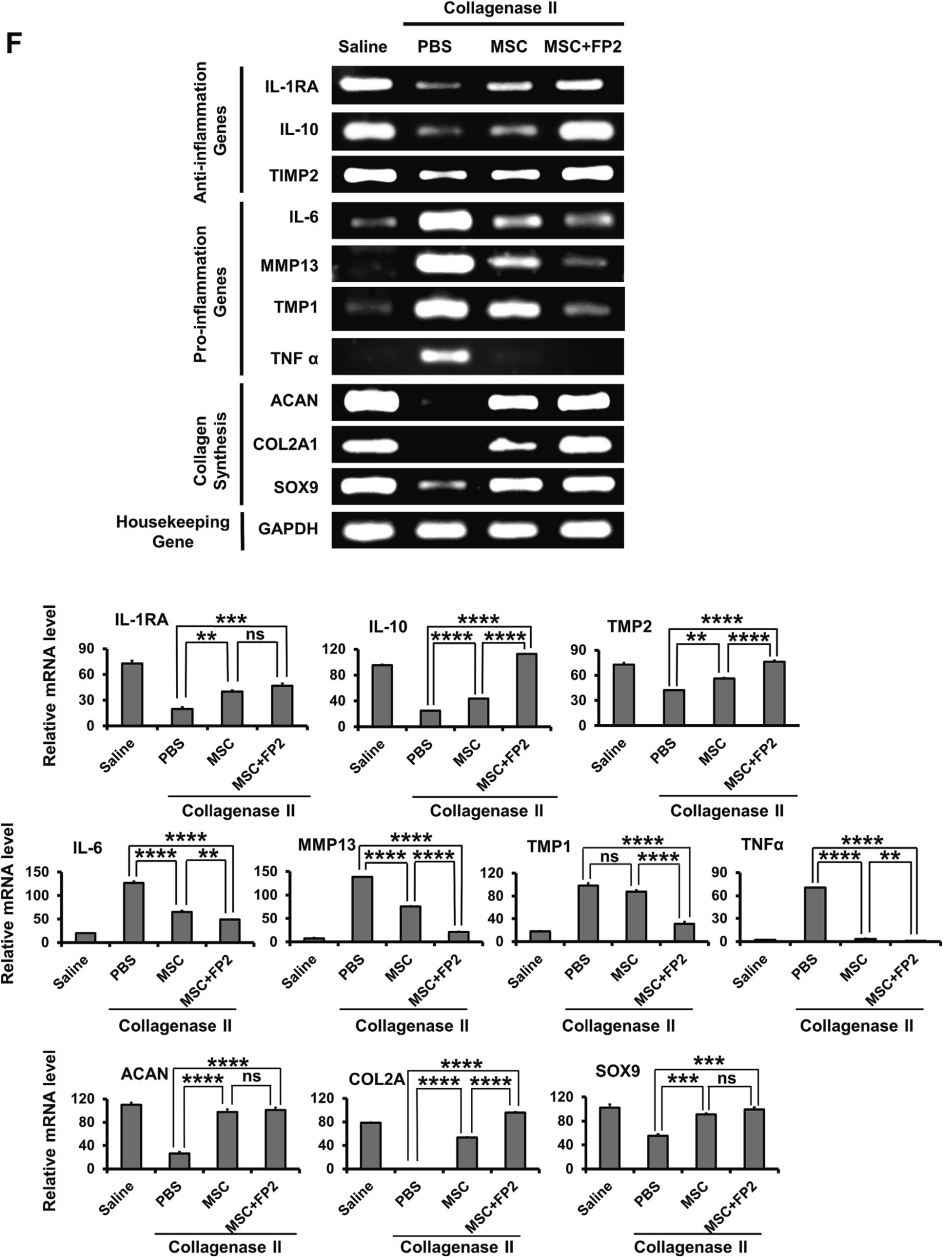
**The in vivo anti-arthritis activity of FP2 in OA mice model**. **(A)** Calibration of knee joint swelling after COL II injection and injection of FP2-cultured cells and control cells on day 3 after OA induction. We performed OA via the intra-articular injection of 6 μl of 13U of COLII that was diluted in saline into the knee joint using a 26G Hamilton syringe. **(B)** Change in the body weights of OA mice after injection of FP2-cultured cells and control cells. **(C)&(D)** Rotarod test for evaluating behavioral alteration in OA mice with and without injection of FP2-cultured cells and control cells. This test is represented by the first round around time (in seconds) (left panels) and failing frequencies (right panel). Data shown in rotarod test are presented saline, PBS, and MSC group **(E)** and saline, PBS, and MSC + FP2 group **(F)**. RT-PCR analysis of anti-inflammatory genes (IL-1RA, IL-10, and TIMP2), pro-inflammatory genes (IL-6, MMP13, TMP1, and TNFα), and collagenase synthesis-related genes (ACAN, COL2A1, and Sox9) in the joints of the mice sacrificed after OA induction and cell injection. GAPDH was used as a control. The lower panels display a graphic representation of the data obtained from the PCR bands, which was quantified using the ImageJ software. *p < 0.05, **p < 0.01, ***p < 0.001, ****p < 0.0001, ns, not significant. **Abbreviation**: ACAN, aggrecan; COL2A1, collagen type II alpha 1; Sox9, SRY (sex determining region Y)-box 9; IL-1RA, interleukin 1 receptor antagonist; TIMP2, tissue inhibitor of metalloproteinase 2; MMP13, matrix metallopeptidase 13; TNF, tumor necrosis factor.

The intra-articular injection of FP2-cultured hWJ-MSCs significantly decreased joint swelling induced by COL II, especially on day 3 after cell injection (Fig. 6**A**). In addition, the mice injected with FP2-cultured cells showed a complete recovery in joint swelling on day 12 post-injection compared with the mice injected with non-FP2-cultured hWJ-MSCs, which showed recovery in joint swelling on day 24 after cell injection (Fig. 6**A**). We did not detect any marked changes in the body weight of the tested mice (Fig. 6**B**).

Next, we confirmed the ability of the FP2-cultured cells to reduce motor dysfunction caused by rotarod-induced pressure and stress on the knee joint. COLII-injected mice did not stay at the top of the rotating rod as long as the saline-treated group showed higher failing frequencies (Fig. 6**C**). This dysfunction was significantly ameliorated by the injection of FP2-cultured hWJ-MSCs starting from day 3 after the cells were injected (Fig. 6**D**). In contrast, the mice that received an injection of non-FP2-cultured hWJ-MSCs did not show a marked improvement in motor dysfunction until day 10 post-injection (Fig. 6**C**).

Similarly, the injection of FP2-cultured cells into the mice significantly reduced the failing frequencies induced by COL-II injection, and they showed a marked reduction in failing frequencies compared to the mice that had been injected with non-FP2-cultured hWJ-MSCs (Fig. 6**D**).

COL-II-induced OA mice showed an alteration in bone phenotype, including loss of articular cartilage (indicative of damage to glycosaminoglycans (GAGs)) and cartilage degeneration (related to proteoglycan levels), which were visualized using Safranin O/fast green and toluidine blue staining (**Supplemental** Fig. 6**B**). Our results showed that FP2-cultured cells improved cartilage regeneration after the degeneration induced by COLII injection (**Supplemental** Fig. 6**B**). We then estimated the OA grades after Safranin O/fast green staining using Osteoarthritis Research Society International (OARSI) scores that grade the degree of rodent cartilage degeneration from minimal (0) to maximal (6) [67]. The injection of FP2-grown cells significantly decreased OA scores compared with the control COLII-injected mice, and the FP2-cultured cells had a better effect than the injection of the control cells (**Supplemental** Fig. 6**C**).

Moreover, we estimated the expression levels of anti-inflammatory genes, pro-inflammatory genes, and collagen synthesis genes in the bone tissue after the mice were sacrificed. Compared with the COL-II-injected mice, mice that received FP2-grown cell transplants had significantly enhanced expression levels of anti-inflammatory genes, namely interleukin-1 receptor antagonist (IL-1RA), IL-10, and tissue inhibitor of metalloproteinase 2 (TIMP2) (Fig. 6**F**).

Furthermore, significant suppression of the expression levels of pro-inflammatory genes, including IL-6, matrix metalloproteinase (MMP) 13, tissue inhibitors of metalloproteinases 1 (TMP-1), and tumor necrosis factor-alpha (TNFα), was detected after the injection of FP2-grown cells (Fig. 6**F**). Interestingly, FP2-cultured cells promoted the expression of genes involved in collagen synthesis including aggrecan (ACAN), collagen type II alpha 1 chain (COL2A1*)*, and SOX9 (Fig. 6**F**). Of note, the injection of FP2-grown cells showed a markedly better effect in ameliorating OA-associated upregulation of pro-inflammatory genes and upregulation of anti-inflammatory and collagen synthesis genes than the effect that was observed after the injection of non-FP2-cultured hWJ-MSC (Fig. 6**F**).

Taken together, the injection of FP2-grown hWJ-MSCs into COLII-injected mice significantly ameliorated OA-associated knee joint swelling, motor ability dysfunction, and histological changes in the joint cartilage, and had a better therapeutic activity against the in vivo OA-associated inflammatory changes compared to the effects observed upon injection of non-FP2-cultured hWJ-MSCs.

## Discussion

Multipotent MSCs are heterogeneous cells that have garnered global attention for their intriguing therapeutic attributes, such as a tendency for multilineage differentiation, the capacity to migrate to the site of damage, and their immunomodulatory and anti-inflammatory functions [89], [90], [91]. The heterogeneity as well as the therapeutic capacity of MSCs are attributed to their source, isolation method, culture media, and biochemical or mechanical cues [92], [93], [94]. There is a wide range of small molecules, growth factors, and proteins that are utilized for the culture and differentiation of stem cells and their applications in tissue regeneration [95], [96], [97], [98]. Growth factors play vital roles in the modulation of various cellular events, including cell survival, proliferation, migration, and differentiation. However, using those growth factors in their native form hold various challenges, such as activation of the immune reaction, carcinogenicity, their liability to be contaminated, and their short half-life [99], [100]. Therefore, the application of peptide mimetics holds various merits that overcome the usage of native or recombinant proteins, including stability, cost-effectiveness, and easily tailored [101], [102].

Boosting the proliferation capacity and obtaining enough cell numbers from the cultured MSCs is an essential step for their clinical application. To this end, we applied a coating method for the expansion of MSCs based on various engineered FGF-2-derived peptide mimetics, including FP1(canofin1), FP2 (yet undefined), FP3 (hexafin2), and FP4 (canofin3), which are all FGFR agonists. We then investigated the proliferation and differentiation potentials of hWJ-MSCs. Previous reports demonstrated the effect of FP1 and FP4 peptides on enhancing the neuronal differentiation of the cerebellar granule neurons and their neuroprotective function [103]. Moreover, the positive in vitro and in vivo impacts of FP3 on boosting neuronal survival have been reported [25], [26]. Of note, there are no reports on the function of FP2, and our study is the first to show the molecular function of FP2 in the growth and differentiation of MSCs.

The application of the peptides in stem cell culture is carried out via various strategies, including plate coating or conjugation with hydrogel or scaffold [95]. Here, we coated the culture plate with the peptides that fused with the MAP, which guarantees the proper orientation of the peptides and avoids any non-specific bindings [22], [95]. Marine mussels, the source of MAP, are rich in lysine and DOPA [28]. DOPA, a mussel-inspired immobilization strategy, can potently bind to any surface via covalent or non-covalent bonds and forms an intermediate layer for further binding of other molecules or peptides [104], [105]. We consider our procedure for peptide coating onto the culture plate a facile and cost-effective immobilization method that is efficient and avoids the complexities of peptide conjugation with scaffolds or hydrogel. However, further study to investigate the synergistic effect of these peptides when conjugated with a scaffold or hydrogel will be an interesting topic.

FGF-2 is a key growth factor and is a member of the receptor tyrosinase kinase (RTK) family, which regulates various cellular functions in various tissues and organs, as well as embryonic development, wound healing, and angiogenesis, have been reported [106], [107]. The positive impact of FGF-2 on MSCs proliferation and differentiation has been reported [12], [14], [108], whereas, some reports have shown that FGF-2 has a negative impact on the differentiation of MSCs [109], [110]. The addition of FGF-2 to stem cell culture media is essential for maintaining their self-renewal capacity, pluripotency, and undifferentiated status [111]. However, the native form of basic FGF (bFGF) is somewhat unstable, which is attributable to the lack of sulfur bonds in bFGF that protect the protein from physical and chemical-associated alteration [112], [113], [114]. To maintain FGF-2 activity for as long as possible, FGF-2 should be added to the culture media periodically and freshly due to its unstable nature, substantially increasing cost. Therefore, various reports have engineered FGF-2 into potent forms to assure the sustained and controlled release of FGF-2, such as protease-resistant or thermally stable forms of FGF-2 or conjugation of FGF-2 with scaffolds [115], [116].

Similarly, we investigated the impact of the engineered FGF-2-derived peptide mimetics on the proliferation kinetics and differentiation of hWJ-MSCs. We screened the effects of the peptides on the growth kinetics of hWJ-MSCs and found that the FP2 peptide with the best activity, which showed higher proliferation in a time-dependent manner (up to day 12) and higher number of CFUs (that indicates that they have a high self-renewal capacity) compared to the other peptides. In our previous study, FP2 peptides did not show any outstanding effect in promoting the alkaline phosphatase activity and the expression level of the stemness markers in hiPSCs [22]. This phenomenon might be ascribed to the differences in the cell lines as multipotent for MSCs and pluripotent cells for hiPSCs, which needs further investigation. Of note, our preliminary screening showed that FP2 had the best activity and FP4 had the least activity, which could be explained by the fact that the energy of the interaction of FP2 with the receptor is higher than that of FP4, as demonstrated in Table 3. However, further in-depth computational analyses need to be performed.

FP2-grown cells showed delayed senescence-associated changes that were shown in the significant decrease in the population of SA-β-gal positive cells compared with the control cells. The senescent cells secrete detrimental products, such as pro-inflammatory cytokines, matrix proteases, and paracrine factors, which have a negative implication on cell proliferation via the induction of cell cycle arrest [117], [118]. The impact of FGF signaling on delaying stem cell senescence has been reported [119], [120]. Moreover, the malfunction of FGF signaling is associated with the development of the aging phenotype in vivo [121], [122], [123]. In sum, we could correlate the capacity of FP2 coating to enhance cell proliferation with its capacity to delay cellular senescence, and future investigations on the in-depth mechanisms of FP2 in delaying cellular senescence in vitro and in vivo are needed.

In our previous study, we screened the potential of various ECM- and FGF-2-derived mimetic peptides for enhancing the adhesion, proliferation, and pluripotency of hPSCs in comparison to Matrigel [22]. Fibronectin (ECM-derived) and canofin peptide (FGF-2-derived) offer superior functionality in boosting hPSC adhesion and pluripotency compared to other test peptides. In contrast, our current study findings show that FGF-2-derived FP2 peptide but not canofin peptide has the best activity in promoting MSCs proliferation, differentiation, and in vivo therapeutic activity. We could attribute the difference in peptide activity between our two studies to the differences in the cell lines as multipotent for MSCs and pluripotent cells for hiPSCs and that needs further investigation in future studies. Notwithstanding, previous reports showed a discrepancy in peptide behavior even for the same line. For instance, Lambshead et al. showed the potent effect of the cyclic peptide c(RGDfK) in enhancing hPSC adhesion [124], whereas Klim et al. demonstrated the negative effect of the same peptide in hPSC culture [125]. Taken together, differential action of the same peptide could be detected between the same or various cell lines.

Sohi et al. demonstrated the potent synergy of FGF-2 peptide when co-immobilized with vitronectin (VN) peptide onto chitosan surface for enhancing the adhesion, growth, and the pluripotency of iPSCs [126]. One of the therapeutic mechanisms of MSCs is their capacity to differentiate from other tissue such as bone or cartilage. FGF-2 is a potent cytokine that boosts the initial stage of bone and cartilage regeneration [122], [127].

FP2-cultured cells showed a marked increase in osteogenic and chondrogenic differentiation capacity, as proven by differentiation-associated staining and the significant increase in the expression level of lineage-related markers. Similarly, a research report by Lee et al. demonstrated the capacity of FGF-2-derived peptides conjugated with a chitosan surface to promote the osteogenic differentiation of h-BM-MSCs [100].

In our study, we demonstrated a marked increase in ITGA6 expression in FP2-grown cells compared to control cells, which is in accordance with a recent report showing a link between ITGA6 and enhanced proliferation, differentiation, and CFU numbers in MSCs [76]. NF-κB is upstream of C-Myb; therefore, we confirmed the increased phosphorylation of IKKα, which is one of the activating kinases for NF-κB. The role of NF-κB in promoting the osteogenic differentiation of human MSCs has been reported [128].

The binding of FGF to FGFRs triggers FGFR dimerization, which results in receptor autophosphorylation [16]. The phosphorylation of the tyrosine residues in FGFR leads to the docking of FRS2α, Src homologous and collagen A (ShcA), and phospholipase-Cg, which results in the stimulation of key signaling pathways, such as the mitogen-activated protein kinase and phosphoinositide-3 kinase (PI3K) pathways [19], [23], [111]. Similarly, our study showed that FP2-cultured cells had increased phosphorylation of the AKT and ERK signaling pathways and that the phosphorylation of AKT was markedly higher than that of ERK. RNA-seq results showed upregulation of PI3K/AKT signaling in FP2-cultured cells compared to control cells. The role of PI3K/AKT signaling in proliferation and differentiation has been reported previously [129], [130], [131], [132], [133]. Our in vitro experiment demonstrated that treating the FP2-grown cells with an AKT inhibitor significantly suppressed their osteogenic and chondrogenic differentiation, whereas treating them with an ERK inhibitor did not show the same activity.

Taken together, we propose that FP2 interacts with FGR1 and activates FRS2α, which leads to phosphorylation of the PI3K/AKT and ERK signaling pathways, as demonstrated by the western blot analysis. RNA-Seq. analysis data showed the upregulation of NF-κB signaling, and we confirmed the higher expression of the IKKα protein in FP2-grown cells than in control cells, which ultimately enhanced the proliferation and differentiation-associated transcription factors. The graphical abstract outlines the proposed mechanism of FP2 in our study.

We then sought to verify the in vivo anti-inflammatory activity of FP2-grown cells by injecting them into a COLII-mediated experimental OA mouse model. The intra-articular injection of FP2-cultured cells markedly reduced OA-associated symptoms in mice, such as joint swelling, abnormalities in gait and balance, and cartilage degeneration, as indicated by a reduction in the OARSI score. Analysis of joint tissue from the injured mice showed significant upregulation of anti-inflammatory genes and downregulation of pro-inflammatory genes, and upregulation of the expression level of collagen synthesis-associated genes after injection of FP2-cultured cells as compared to the injection of control MSCs. RNA-seq data analyses using a protein–protein interaction network showed downregulation of the TNF signaling pathway- and rheumatoid arthritis-associated genes in FP2-cultured cells versus control cells. Moreover, the expression level of inflammatory-associated cytokines and interleukins genes including CCL20, and IL-6 were downregulated in FP2-cultured stem cells, which might explain the in vivo anti-arthritic activity of FP2 peptide. FGF signaling regulates the growth, development, and homeostasis of joint-related cells including articular chondrocytes, synovial cells, and osteogenic cells, which are crucial to maintaining the health and functional state of joints [134], [135]. More details on the effects of FGFs on the modulation of cartilage development and osteoarthritis therapy have been reviewed in detail elsewhere [136].

Finally, we applied state-of-the-art computational tools, such as AI-based AlphaFold2 and CABS-dock tools, and three-dimensional (3D) structure analysis for prediction and visualization of how these peptides could interact with the receptor and for the explanation of peptides potent in vitro and in vivo activities. Through these analyses, we could detect a different behavior of the peptides in their interaction with the receptor, and we could also detect a difference in the nature of the interaction bonds. Further computational analyses using the current study peptides, together with designing other growth factor-derived peptides and comparing their activities in modulating stem function, need to be elaborated in further study. We could demonstrate the biological function of FP2 in WJ-MSCs via several in vitro and in vivo experiments. However, the precise degree of the peptide’s functionalization on the culture plate and its correlation to the peptide's activity need to be explained in future studies.

## Conclusion

Our study unraveled novel functions of FGF-2 derived peptides mimetics in modulating hWJ-MSCs biological functions. In particular, we demonstrated the high activity of FP2 to generate highly proliferating hWJ-MSCs with improved self-renewal and differentiation capacities compared to the other peptides. We also verified the involvement of FRS-2α after FGFR1 activation in the phosphorylation of PI3K/AKT and NF-κB signaling pathways, which are implicated in FP2-mediated enhanced proliferation and differentiation of hWJ-MSCs. We also are planning on exosome isolation from FP2-hWJ-MSCs and verifying the therapeutic application of FP2-hWJ-MSC-derived EVs for the treatment of inflammatory diseases.

Various therapeutic peptides are undergoing preclinical research and clinical development, and more than 80 therapeutic peptides have made it to the market on a global scale [137]. Further research work needs to be implemented for the utilization of FGF-2-derived peptides for clinical applications and bone disease therapy markets. Collectively, our study provides a novel peptide-based niche for efficiently producing MSCs for bone and cartilage regeneration and arthritis therapy. However, more detailed computational analyses are still needed to demonstrate the crosslink between the FGF-2-derived peptides/FGFR interaction and the biological functions in stem cell-based bone regeneration.

## Funding

National Research Foundation (NRF), funded by the Korean Government (Ministry of Education, Science, and Technology), Grant number: 2019M3A9H1030682 and Korean Fund for Regenerative Medicine (KFRM) grant funded by the Korean government (the Ministry of Science and ICT and the Ministry of Health & Welfare) Grant number: 22B0502L1-01. SK acknowledges financial support from the National Science Centre, Grant number: 2021/40/Q/NZ2/00078.

## Data and materials availability

All data needed to evaluate the conclusions in the paper are present in the paper and/or the Supplementary Material.

## Compliance with ethics requirements

All animal experimental procedures were approved by the Institutional Animal Care and Use Committee (IACUC) at Konkuk University (approval no.: KU20127).

## CRediT authorship contribution statement

**Soo Bin Lee:** Conceptualization, Methodology, Software, Writing – original draft. **Ahmed Abdal Dayem:** Conceptualization, Methodology, Investigation, Writing – original draft. **Sebastian Kmiecik:** Software, Formal analysis. **Kyung Min Lim:** Methodology, Visualization. **Dong Sik Seo:** Visualization, Formal analysis. **Hyeong-Taek Kim:** Visualization, Formal analysis. **Polash Kumar Biswas:** Methodology, Visualization. **Minjae Do:** Methodology, Visualization. **Deok-Ho Kim:** Methodology, Visualization. **Ssang-Goo Cho:** Conceptualization, Methodology, Writing – original draft, Project administration, Funding acquisition, Supervision.

## Declaration of Competing Interest

The authors declare that they have no known competing financial interests or personal relationships that could have appeared to influence the work reported in this paper.
