# Supplementary material for: Efficient improvement of the proliferation, differentiation, and anti-arthritic capacity of mesenchymal stem cells by simply culturing on the immobilized FGF2 derived peptide, 44-ERGVVSIKGV-53
Source: J Adv Res. 2023 Sep 28;62:119–41. doi: 10.1016/j.jare.2023.09.041 (PMC11331723; doi:10.1016/j.jare.2023.09.041)
Supplement: Supplementary data 1 [file mmc1.docx]

Supplementary Materials for

Efficient improvement of the proliferation, differentiation, and anti-arthritic capacity of mesenchymal stem cells by simply culturing on the immobilized FGF2 derived peptide, 44-ERGVVSIKGV-53

Soo Bin Lee ^a,1^, Ahmed Abdal Dayem ^a,1^, [Sebastian Kmiecik](https://sciprofiles.com/profile/253609) ^b^, Kyung Min Lim ^a^, [Dong Sik Seo](https://www.sciencedirect.com/science/article/pii/S1873506120300027#!) ^c^, Hyeong-Taek Kim ^c^, Polash Kumar Biswas ^a^, Minjae Do^d^, Deok-Ho Kim^d^, Ssang-Goo Cho ^a,^*

^*^Corresponding author. E-mail addresses: ssangoo@konkuk.ac.kr (S-G.C.).

**This PDF file includes:**

Figs. S1 to S7

**
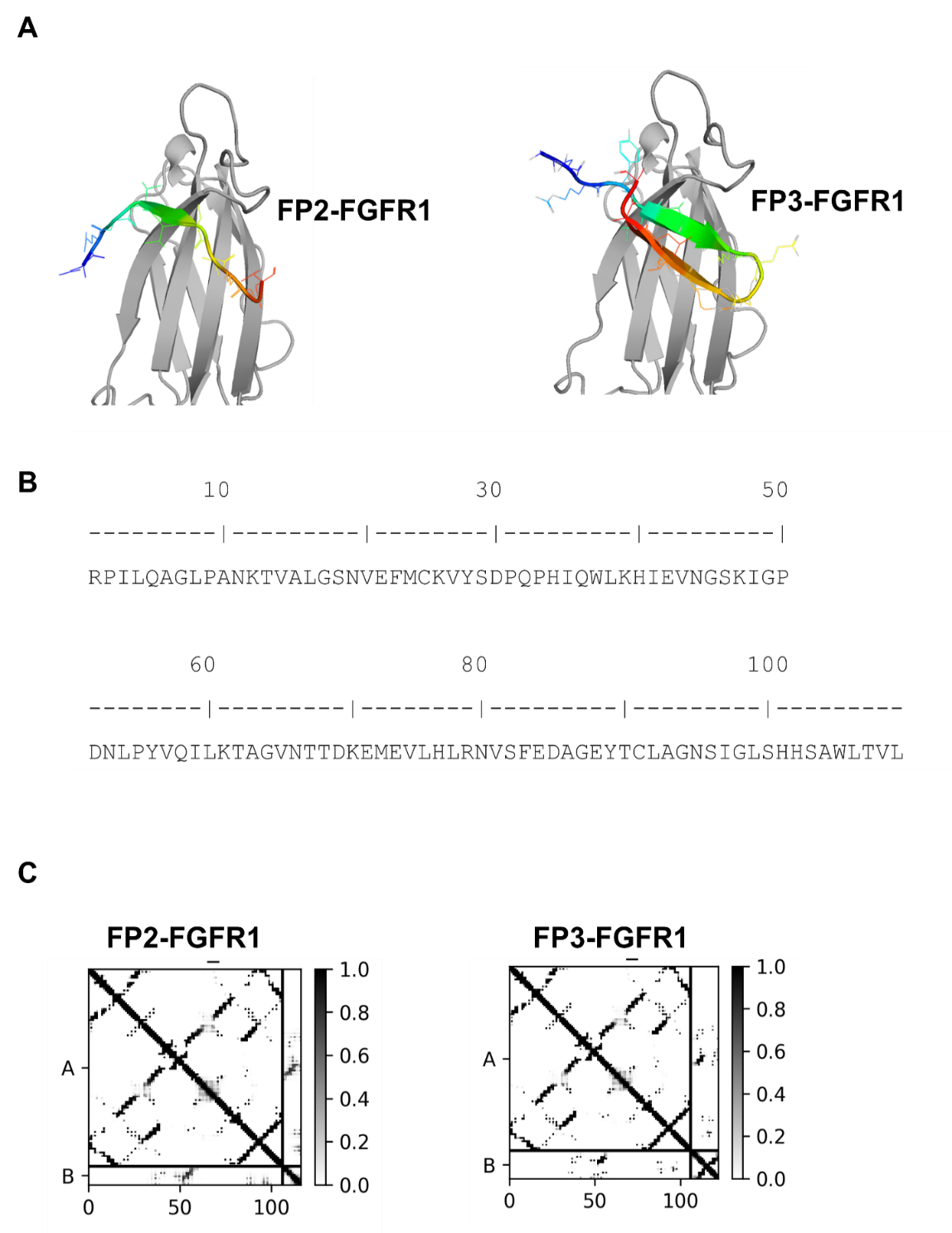
**

**Supplemental Fig. 1. Top-ranked AlphaFold2 predictions of FP2-FGFR1 and FP3-FGFR1 complexes. (A)** Peptides are colored from the N- (blue) to C-terminus (red), and the FGFR1 C-terminal domain is shown in gray. The presented binding modes were characterized using contact maps, which are shown in Figure S1 in the Supplementary Information. **(B)** Numbering of FGF2 domain residues. The sequence of the C-terminal domain of FGF2 protein used in the AlphaFold structure prediction. Note that the Arg-1 residue in the sequence above corresponds to the Arg-254 residue in the 1CVS chain C PDB file. **(C)** Predicted contacts for FP2 and FP3 peptide complexes with an FGFR1 C-terminal domain. A corresponds to the FGF2 domain and B corresponds to a peptide. Both peptides FP2 and FP3 were predicted to bind to the same FGFR1 binding site.


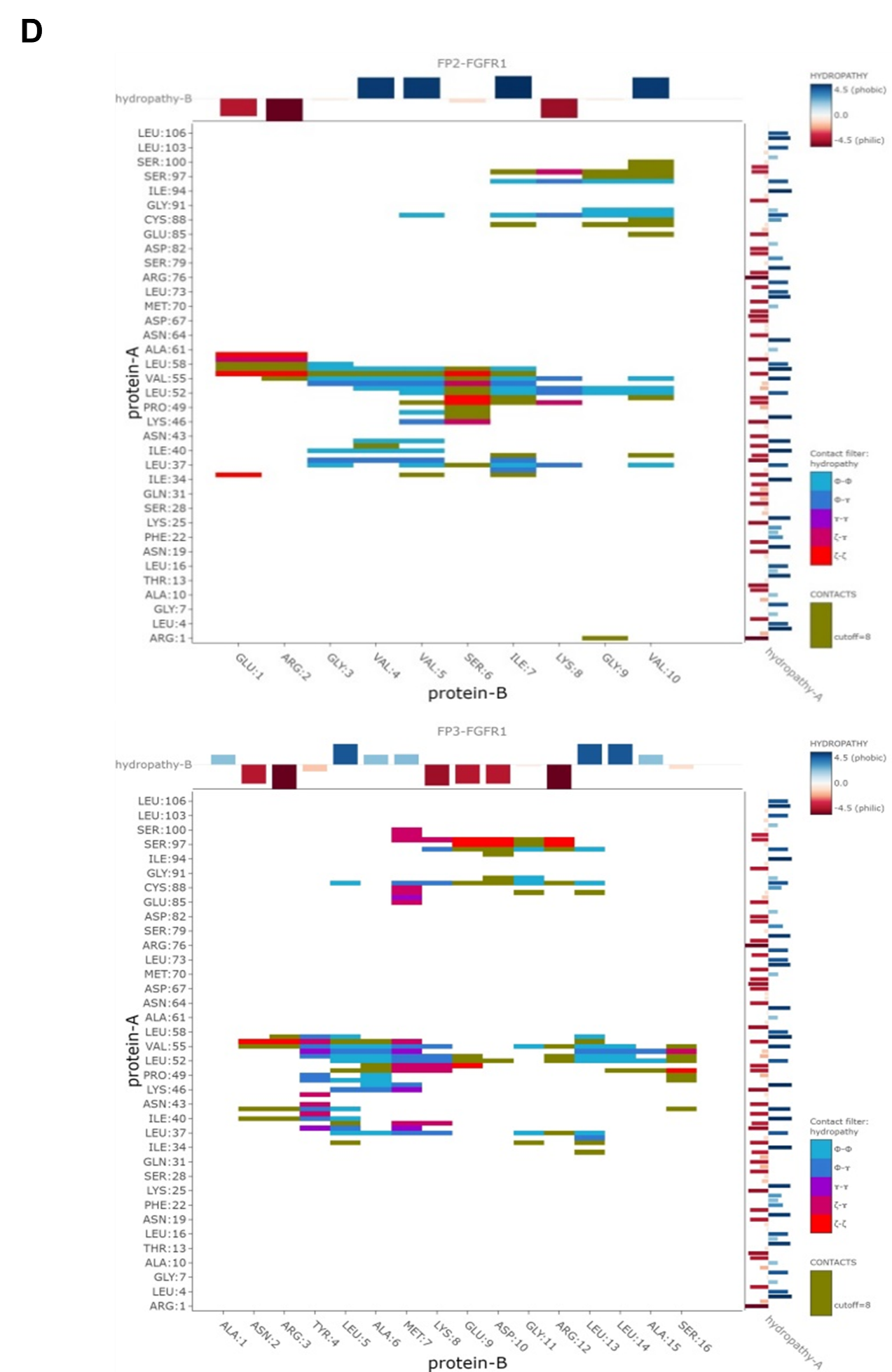


**Supplemental Fig 1. (D) Contact maps for the interaction interfaces of** **FP2-FGFR1 and FP3-FGFR1 predictions.** Contacts are colored according to hydropathy scale (hydrophobic in blue, hydrophilic in red). Images were prepared using Mapiya web server ([doi.org/10.1093/nar/gkac307](https://doi.org/10.1093/nar/gkac307)).


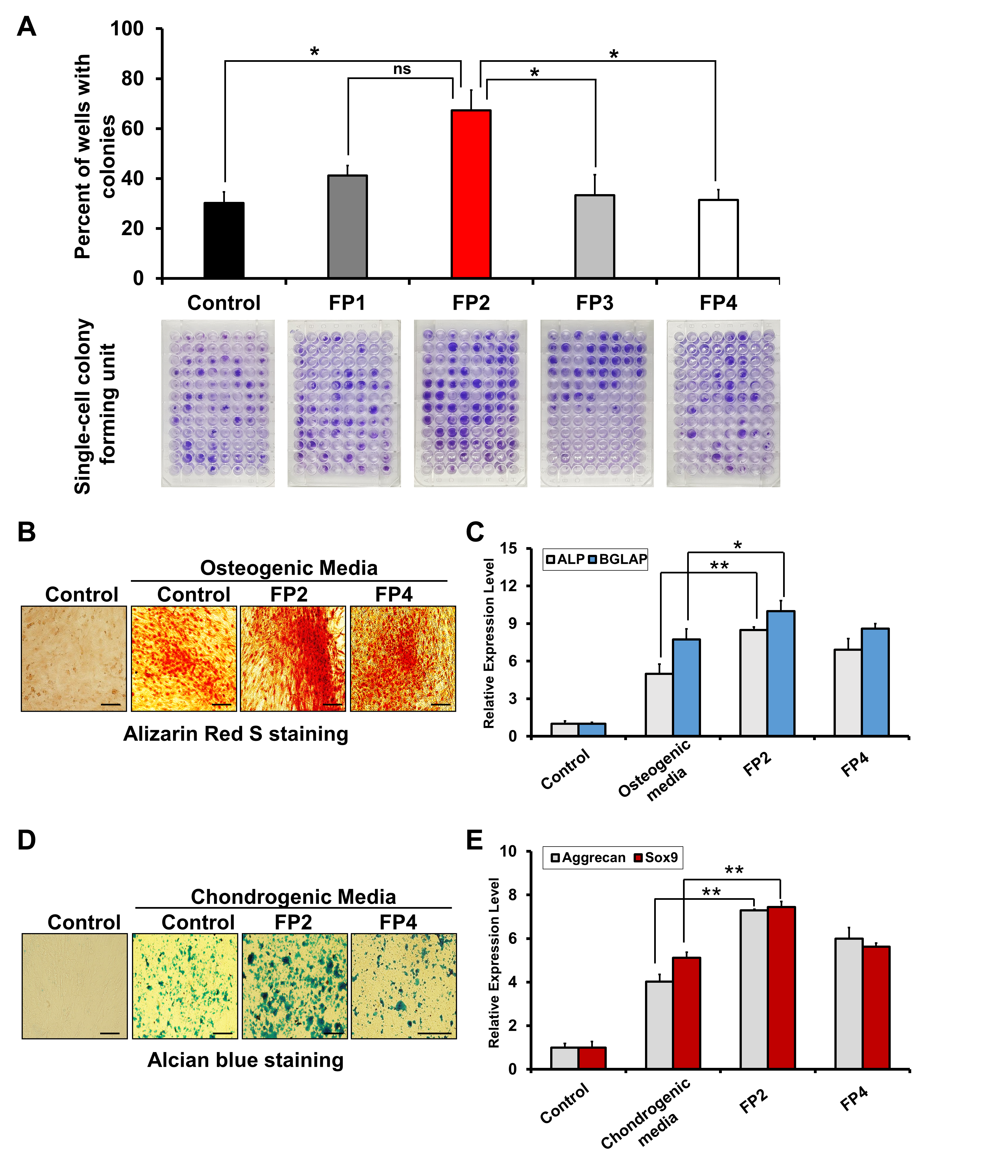


Supplemental Fig. 2. Screening the impacts of FGF2-derived peptide mimetics on the proliferation, CFU capacities, differentiation hWJ-MSCs characterization (A) Culturing of MSCs onto FP2-coated plate showed the best CFU capacity, as confirmed by crystal violet staining. FP2-cultured cells are superior to FP4-cultured cells in osteogenic differentiation ability, as shown by the strong of alizarin red S staining. (B) and the increase in the expression level of two osteogenesis-associated genes, namely ALP and BGLAP using qPCR analysis (C). The chondrogenic differentiation was also better in FP2-cultured cells, as shown by higher intensity of alcian blue staining (D) and the expression level of chondrogenic differentiation-associated genes including aggrecan and Sox9 using qPCR analysis (E). Scale bar, 200 μm. Data are presented as mean ± SEM. For multiple comparisons of groups, a one-way analysis of variance (ANOVA) was performed followed by post hoc tukey’s multiple comparison, (n=3) **p* < 0.05, ** *p* < 0.01, ns, not significant.


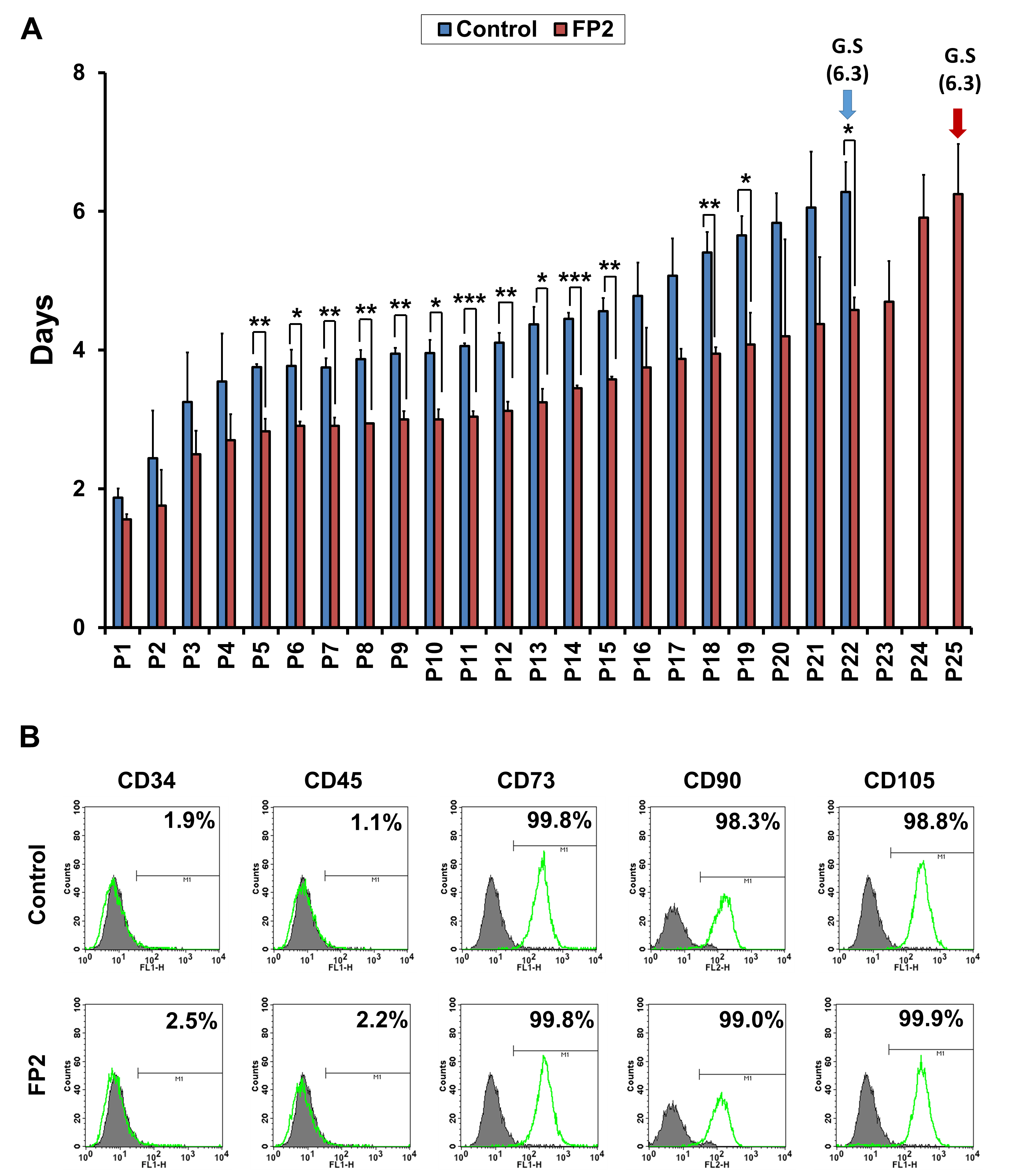


Supplemental Fig. 3. The impacts of FP2 on doubling Times and surface markers expression of the hWJ-MSCs

**(A)** FP2 enhances the proliferation of hWJ-MSCs up to P25, whereas hWJ-MSCs without FP2 stopped growing at P22. Cell proliferation analysis was carried out via cell counting using a hemocytometer after staining with 0.4% trypan blue solution. Data are presented as mean ± SEM. All experiments were performed for three independent times, and Statistical significance was determined using Two-tailed t test at each passage (n=3), **p* < 0.05, ***p* < 0.01, ****p* < 0.001. **(B)** FP2 coating did not cause changes in the expression level of five cell surface markers, namely CD34, CD45, CD73, CD90, and CD105, as determined by FACS analysis.


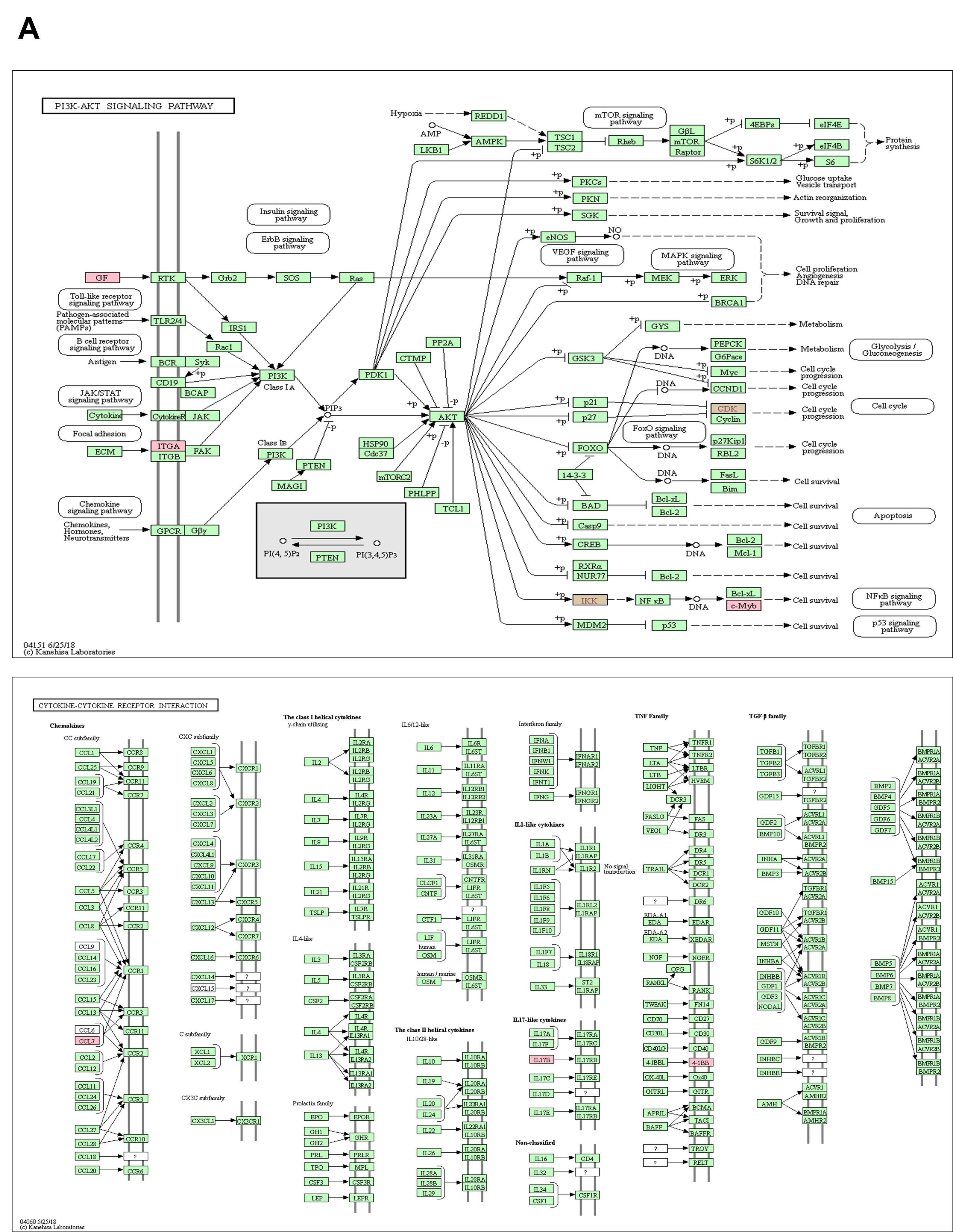

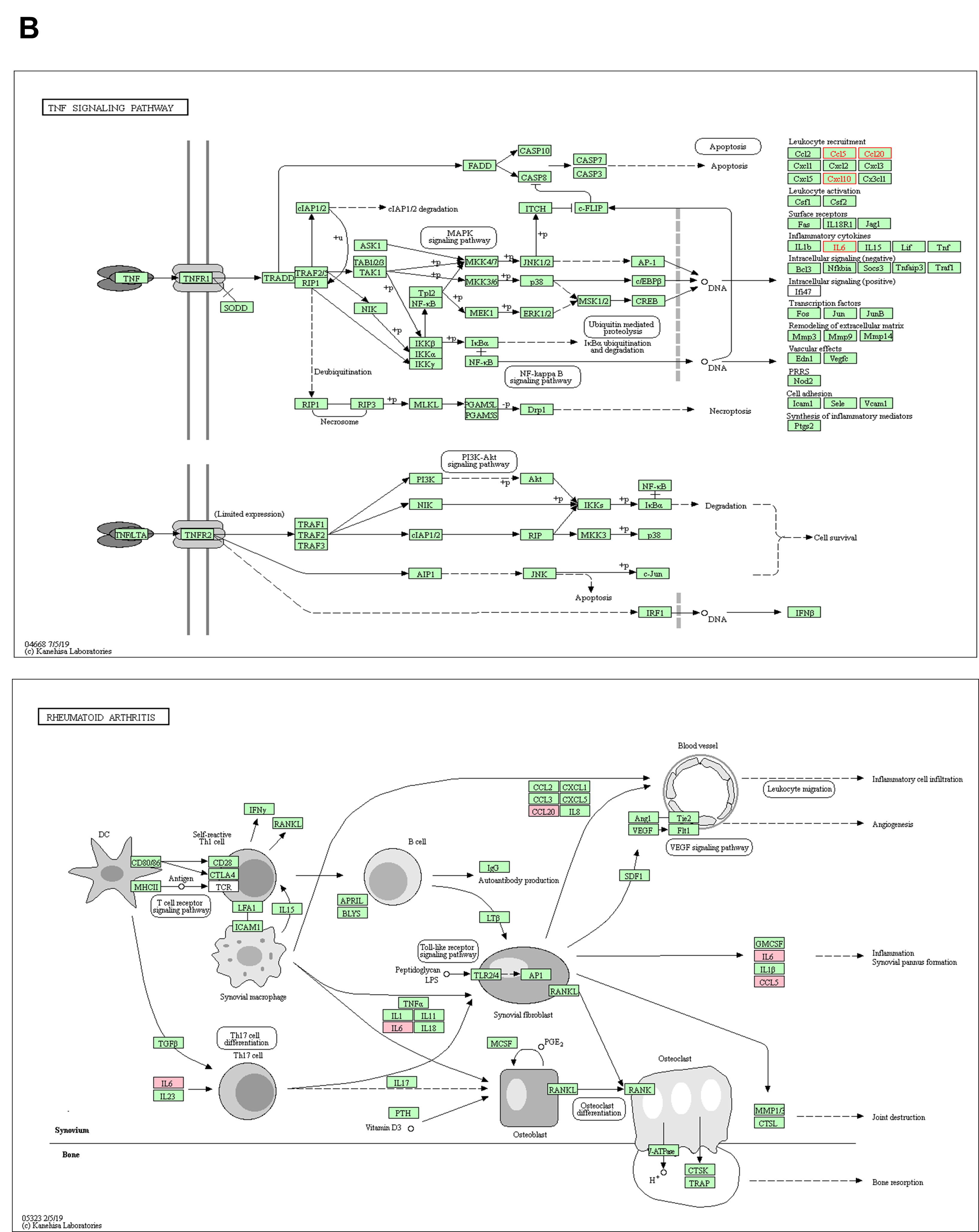


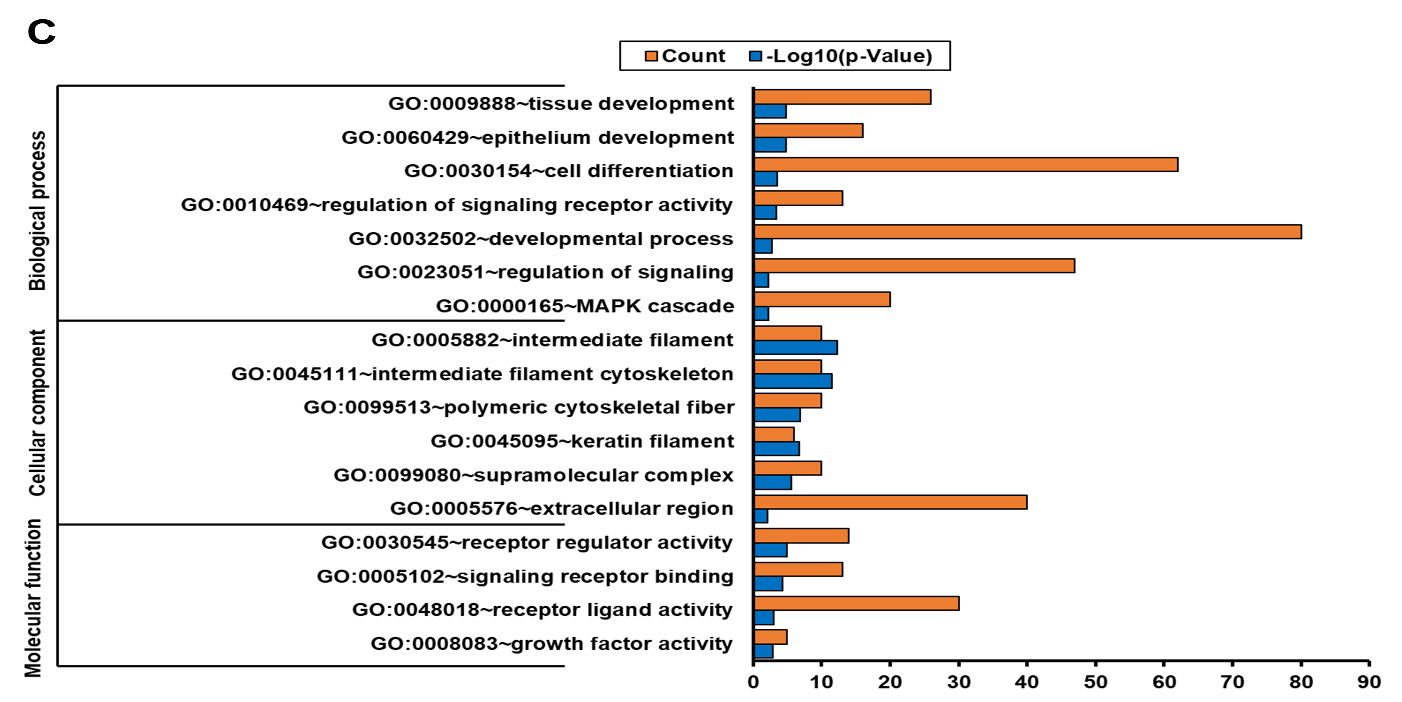


**Supplemental Fig. 4.** Analysis of the differentially expressed genes (DEGs) in FP2-cultured cells versus control cells. KEGG analysis identifying upregulated **(A)** and downregulated **(B)** signaling pathways in FP2-cultured cells versus control cells. **(C)** Functional enrichment of DEGs based on gene ontology (GO) categorization, including significantly enriched terms for BP, CC, and MF. The analysis of these enrichments was carried out using the DAVID software. The x-axis represents the ID and its equivalent category. The y-axis represents –log10 (p-Value) and the number of genes for each term.


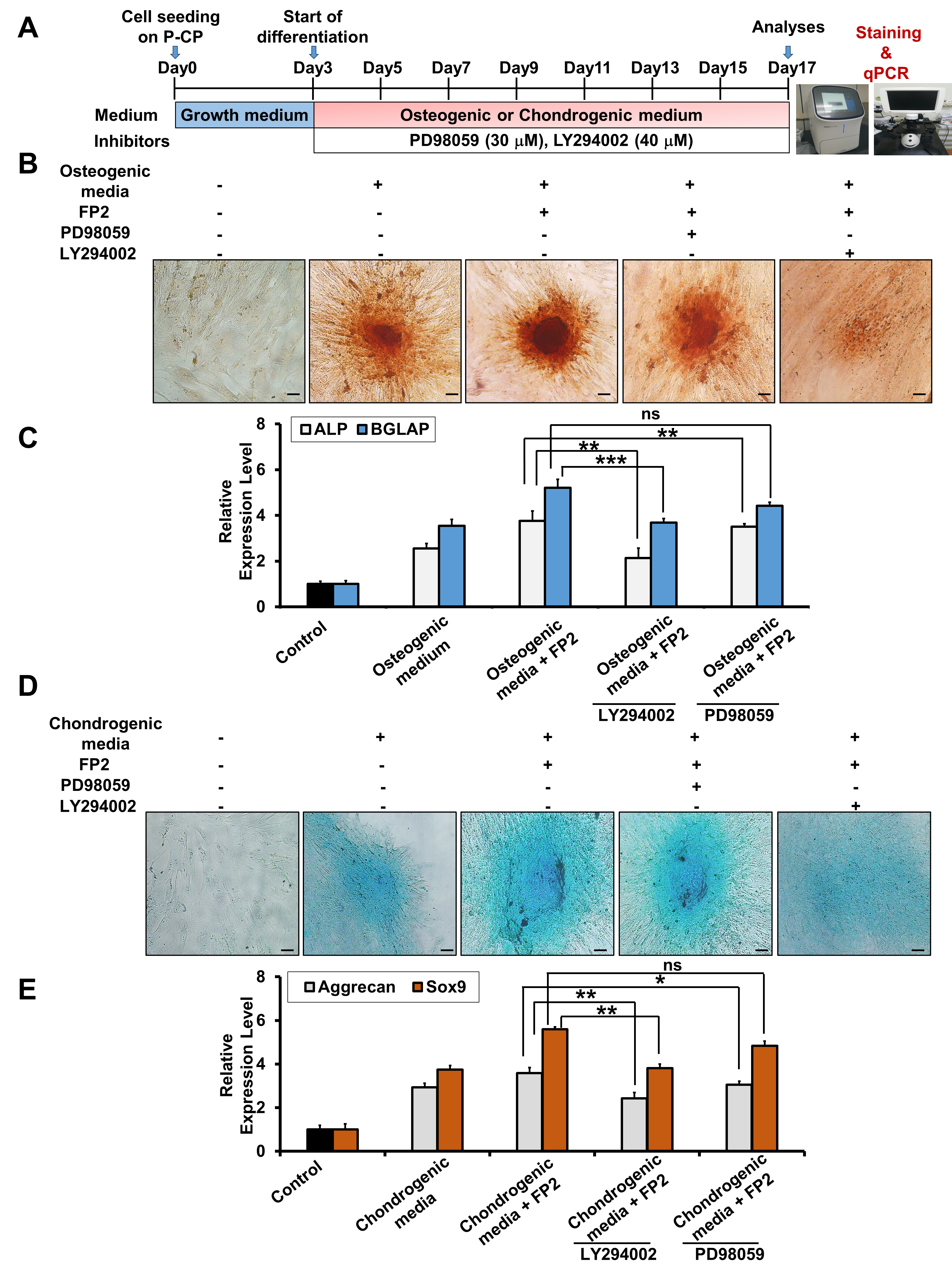


Supplemental Fig. 5. Effects of AKT and ERK signaling inhibitors on the FP2-mediated enhanced differentiation of hWJ-MSCs (A)Schematic diagram depicting the experimental schedule for differentiation of hWJ-MSC in the presence of ERK and AKT inhibitors. The impacts of PD98059 and LY294002 on osteogenic differentiation as shown by alizarin red staining (B) and the expression levels of osteogenesis-related genes (ALP and BGLAP) (C) and genes related to chondrogenic differentiation as shown by the Alcian blue staining (D) and the expression level of chondrogenic differentiation-associated genes (aggrecan and Sox9) (E). Scale bar, 200 μm. Data are presented as mean ± SEM. For multiple comparisons of groups, a one-way analysis of variance (ANOVA) was performed followed by post hoc tukey’s multiple comparison, (n=3) **p* < 0.05, ***p* < 0.01, ****p* < 0.001, ns, not significant. Abbreviation: P-CP, peptide-Coated Plate.


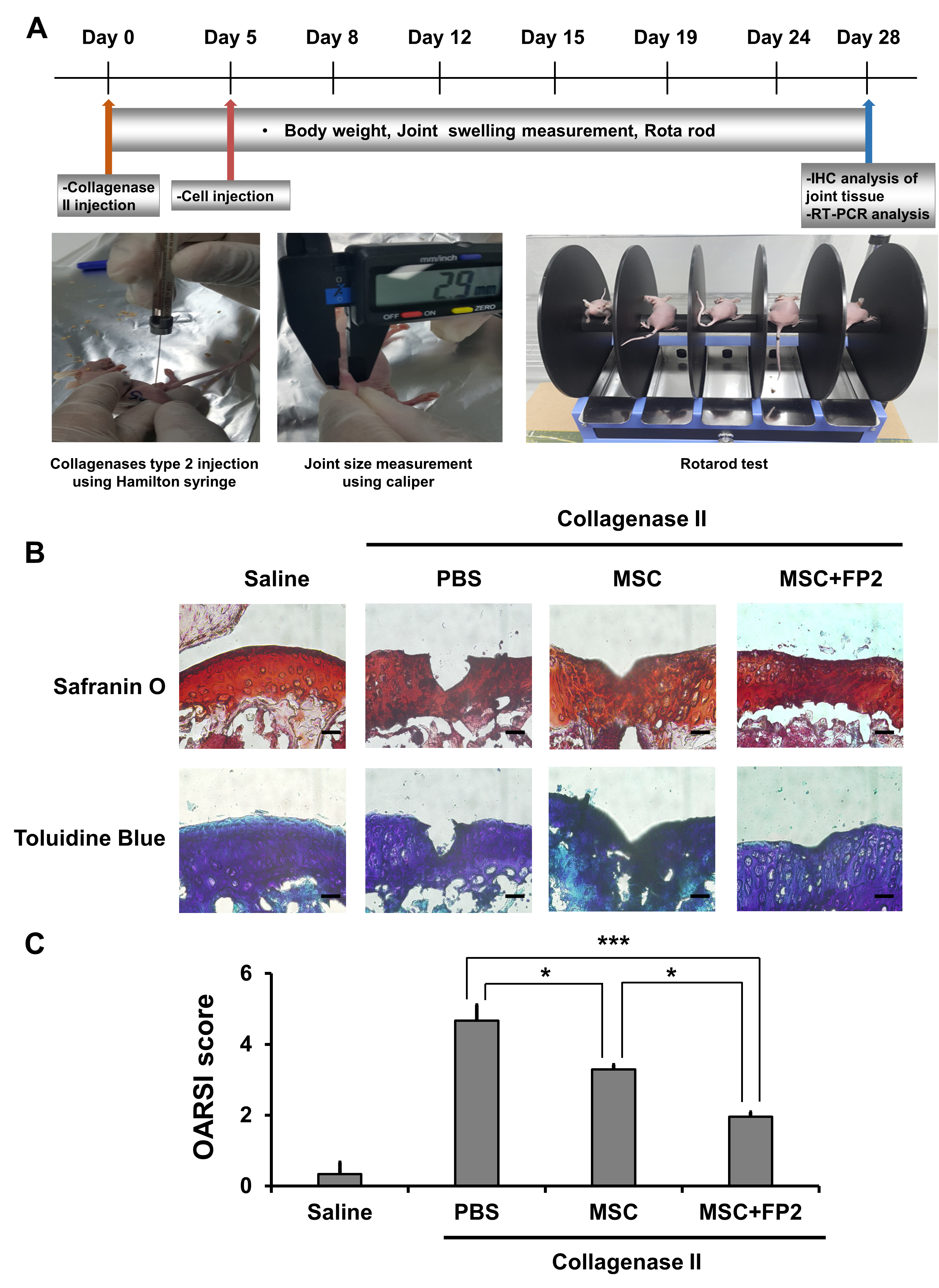


**Supplemental Fig. 6. The in vivo anti-arthritis activity of FP2 in experimental osteoarthritis (OA) mice model (A)** Schematic diagram showing the experimental schedule for induction of OA in mice, cell injection, and analytical processes. The lower panel illustrates the procedure for intra-articular injection of COLII, joint swelling calibration using a caliper, and a rotarod machine with mice on top. **(B)** Histological analyses for visualizing the pathological changes in OA mice joints with and without injection of FP2-cultured cells and control cells, which used safranin O and toluidine blue staining. Scale bar, 50 μm. **(C)** OARSI scores indicating the degree of joint degeneration based on Safranin O/fast green staining. Data are presented as mean ± SEM. For multiple comparisons of groups, a one-way analysis of variance (ANOVA) was performed followed by post hoc tukey’s multiple comparison (n=3), **p* < 0.05, ****p* < 0.001. Abbreviation: COLII, Type II collagenase; OARSI, Osteo Arthritis Research Society International.


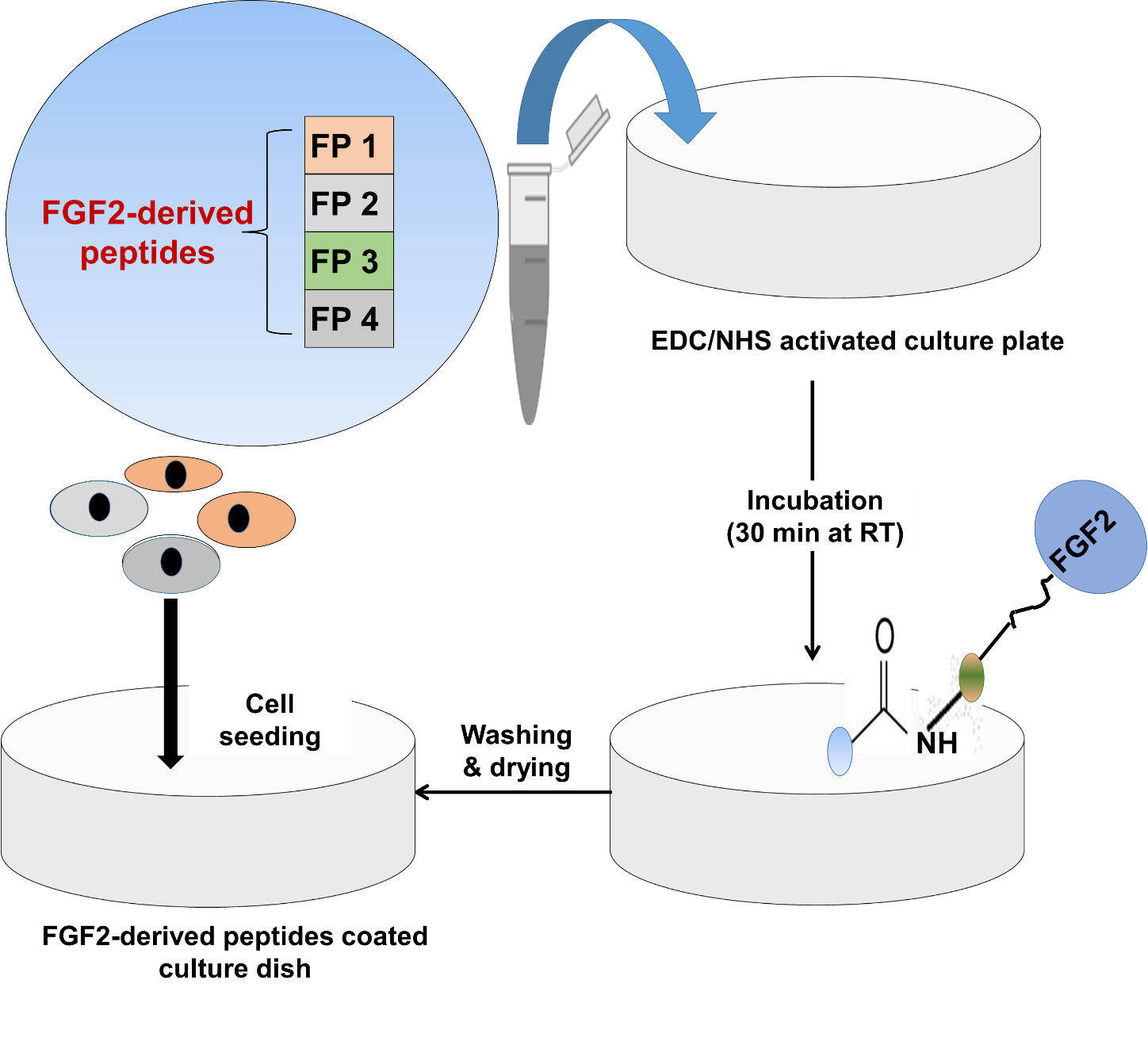


**Supplemental Fig. 7. FGF2-derived peptides coating procedure.** The schematic diagram shows that the peptide coating method was performed via the pre-activation of the culture plate using the EDC/NHS solution. The activated plates were then coated with MAP-bound FGF2-derived peptides and seeded with the cells.
